# Changes in the contributions of risk factors to under-five mortality in low- and lower-middle-income countries (1997–2022): an analysis of Demographic and Health Survey data

**DOI:** 10.1007/s12519-025-00912-8

**Published:** 2025-05-10

**Authors:** Bereket Kefale, Jonine Jancey, Amanuel T. Gebremedhin, Gavin Pereira, Gizachew A. Tessema

**Affiliations:** 1 Curtin School of Population Health, Curtin University, Perth, Western Australia Australia; 2 Department of Reproductive Health, School of Public Health, Wollo University, Dessie, Ethiopia; 3 enAble Institute, Curtin University, Perth, Western Australia Australia; 4 School of Nursing and Midwifery, Edith Cowan University, Perth, Western Australia Australia; 5 School of Medical and Health Sciences, Nutrition and Health Innovation Research Institute, Edith Cowan University, Perth, Western Australia Australia; 6 School of Public Health, University of Adelaide, Adelaide, South Australia Australia

**Keywords:** Antenatal care, Breastfeeding, Changes, Contributions, Low- and lower-middle-income countries, Risk factors, Under-five mortality

## Abstract

**Background:**

Under-five mortality (U5M) is a critical public health challenge in low- and lower-middle-income countries (LLMICs), where over 90% of global deaths occur. Despite progress, the changing contributions of risk factors to U5M in LLMICs remain unexplored.

**Methods:**

We analysed Demographic and Health Survey (DHS) data from 24 LLMICs across two periods: 1997–2005 and 2016–2022. We included 139,890 live births in 1997–2005 and 319,034 in 2016–2022. A mixed-effects robust Poisson regression model with a log link function was employed to identify risk factors of U5M in each period. Population-attributable fractions (PAFs) were calculated and compared to investigate changes in the contributions of risk factors over time.

**Results:**

U5M attributable to never having been breastfed increased by 15.5 percentage points (95% CI 8.6, 22.9), early maternal age at birth (< 20 years) by 5.4 percentage points (95% CI 3.1, 5.7), and plural births by 1.2 percentage points (95% CI 0.4, 1.8). U5M reductions attributable to maternal secondary education were increased by 5.5 percentage points (95% CI 0.4, 11.0) and tertiary education increased by 2.6 percentage points (95% CI 1.6, 4.2). However, U5M reductions associated with 1–3 antenatal care (ANC) visits decreased by 7.2 percentage points (95% CI 2.4, 11.7).

**Conclusions:**

﻿The main contributors of U5M in LLMICs were never breastfeeding, short birth intervals (<33 months), ANC uptake, higher maternal education (secondary and tertiary), advanced maternal age at birth (≥35 years),  early maternal age at birth (<20 years), very small infants at birth, male sex, plurality, and single motherhood. The contributions of risk factors to U5M have changed over time. Interventions need to prioritise promoting breastfeeding, enhancing maternal education and increasing ANC uptake, and addressing other significant contributors to U5M.

**Graphical abstract:**

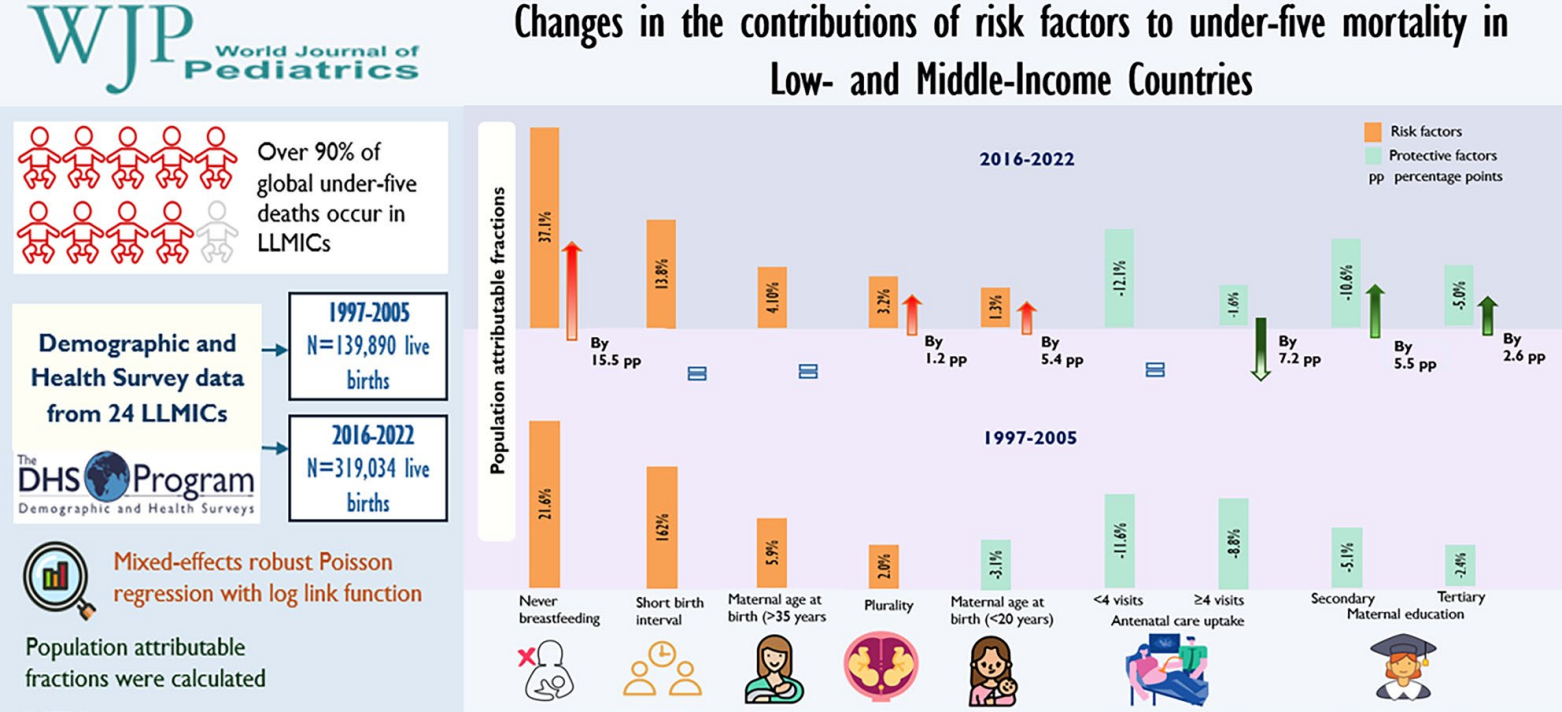

**Supplementary Information:**

The online version contains supplementary material available at 10.1007/s12519-025-00912-8.

## Introduction

Under-five mortality (U5M) refers to the death of a child between birth and the age of five and is a critical indicator of a population’s health and well-being [1, 2]. Global efforts have driven substantial progress in reducing U5M over the past three decades, achieving a 60% reduction, with rates reaching 37 deaths per 1000 live births in 2022 [3].

This significant progress is partly attributed to the coordinated global, regional, and local health and development initiatives targeting key interventions in child health and well-being [4–8]. In 2000, the United Nations Millennium Declaration established the Millennium Development Goals (MDGs), with MDG 4 aiming to decrease the under-five mortality rate (U5MR) by two-thirds between 1990 and 2015 [5]. This led to significant political commitment and investment in maternal and child health interventions, including improvements in maternal and child health services, treatment of common childhood illnesses, expansion of immunisation, promotion of breastfeeding and nutrition, and enhancements in water, sanitation, and hygiene [9–11].

Building on the momentum of the MDGs, the Sustainable Development Goals (SDGs), adopted in 2015, further reinforced the global commitment to improving child health and survival. SDG target 3.2 aims to reduce under-five deaths, specifically to U5MR of ≤ 25 deaths per 1000 live births by 2030 [4]. Achieving this ambitious target has required intensive efforts to scale up essential maternal and child health interventions [12–14], while also implementing a more comprehensive, interconnected approach to address the broader social, economic, and environmental determinants of child health, such as poverty, education, gender equality, and climate change. As a result, considerable progress has been made in various domains, contributing to the decline in child mortality. These include advancements in medical technologies and healthcare interventions, expansions in health research and innovation, improvements in educational attainment, infrastructure development, economic growth, poverty reduction, and broader societal changes like urbanisation [15, 16].

However, the world has also been facing a range of catastrophic public health emergencies and humanitarian crises that have had substantial impacts on child health outcomes. Emerging crises, including human immunodeficiency virus (HIV/AIDS) [17], coronavirus disease 2019 (COVID-19) [18, 19], armed conflicts [20], and climate change [21, 22] have collectively undermined access to essential maternal and child health services, disrupted routine immunisation programs, and exacerbated food insecurity and the spread of infectious diseases.

Consequently, despite significant strides, 4.9 million children are still dying annually from mainly preventable causes in 2022 [3]. Nearly half of under-five deaths are due to infectious and vaccine-preventable diseases [23]. U5M remains uneven across regions, with low- and lower-middle-income countries (LLMICs) disproportionately affected. In 2022, the U5MR was 64 per 1,000 live births in low-income countries (LICs) and 44 per 1000 in lower-middle-income countries (LMICs), which were respectively, 13 and 9 times higher than those in high-income countries (HICs). Over 90% of global under-five deaths occur in LLMICs, emphasising the need for global and domestic investments in addressing child mortality in these regions [3].

Given the advancements and challenges that have occurred, the impact of risk/protective factors on U5M may have shifted over time. While several studies [24–26] identified a range of risk factors affecting U5M at a particular period, to date, no study has explored the changes in the attributable contributions of risk/protective factors to U5M in LLMICs. Thus, this study aimed to examine the changes in the contributions of risk factors to U5M in LMICs from 1997 to 2022. Understanding these changes can help guide targeted interventions and resource allocation to reduce preventable U5M in these high-burden regions.

## Methods

### Data sources

We used Demographic and Health Survey (DHS) data from 24 LLMICs [27]. The DHSs are nationally representative household surveys that provide accurate and reliable data for a wide range of monitoring and impact-evaluating indicators in the areas of population, health, and nutrition [28]. The DHSs are conducted regularly using similar methodologies and instruments across multiple countries. Since the inception of the program, more than 380 DHSs have been carried out in over 90 countries, and DHS data are publicly available [29].

### Inclusion criteria

We included DHS surveys conducted during the early phases of the MDGs (1997–2005) and the most recent available data during the SDG period (2016–2022), to allow examination of the changes in the contributions of risk factors over at least 15 years. We have included 24 countries with DHS-collected data for both the first and second periods (Supplementary Table 2).

### Ethical considerations

This study is a secondary analysis of publicly available data from the DHS program; therefore, ethical approval was not required. An authorisation letter was obtained to access the data for this research (Supplementary Text S1).

### Sampling procedure and population

The DHS used a stratified two-stage cluster sampling technique to select study participants. Samples were first stratified by geographic region, and then further stratified within each region by urban and rural areas. In the first stage, primary sampling units were chosen with probability proportional to size within each stratum; these primary sampling units corresponded to census enumeration areas and constituted the survey clusters. In the second stage, a comprehensive household listing was established within each of the selected clusters, and then a fixed number of households, usually 25–30, were chosen through a systematic random sampling method. Additional details about the sampling procedure used in DHSs can be found elsewhere [29].

**Fig. 1:**
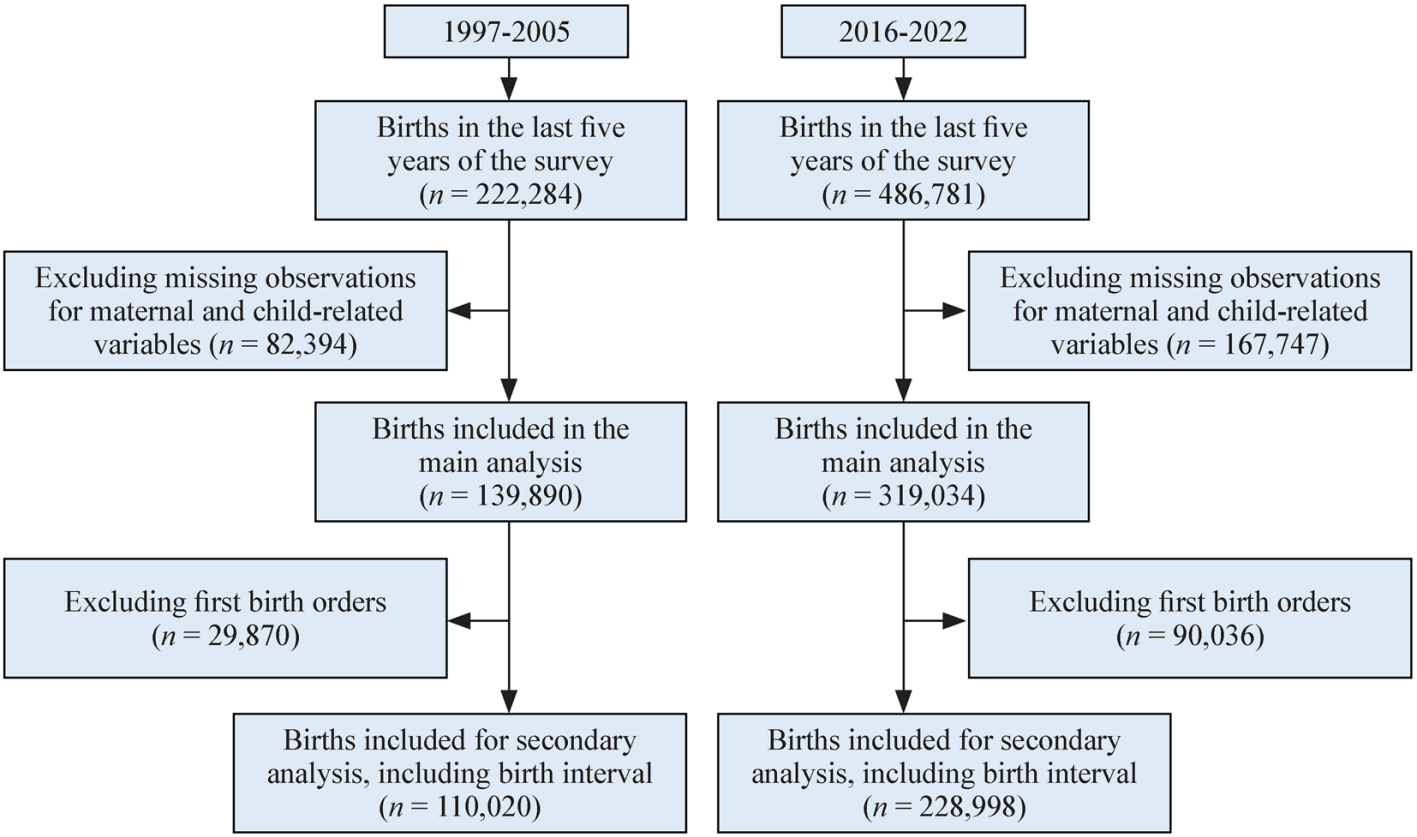
Flow chart showing samples included in the analysis (weighted). The Demographic and Health Survey data have no significant missing; however, certain variables such as antenatal care uptake were collected only for the most recent births

In this study, we used the DHS birth record data during the periods 1997–2005 and 2016–2022. We included only women’s reported births in the last five years before each survey. A total of 222,284 live births were recorded during 1997–2005, while 486,781 live births were recorded during 2016–2022. After excluding births with missing observations for maternal and child-related factors, the final weighted sample included 139,890 live births during 1997–2005 and 319,034 live births during 2016–2022. Additionally, for our secondary analysis accounting for birth interval, we further excluded firstborn births, yielding a weighted sample of 110,020 live births during 1997–2005 and 228,998 live births during 2016–2022 (Fig. 1).

### Measurement of variables

The outcome variable for this study was U5M, defined as the death of a child within five years of birth [29]. The independent variables were classified into the following categories: socio-demographic factors, maternal reproductive and health service-related factors, child factors, and household factors. These factors were selected based on a previous umbrella review [30] and other relevant studies [24, 25, 31], as well as available data in the DHS datasets. Socio-demographic factors included maternal age, maternal educational status, marital status, and place of residence. Maternal reproductive and health service-related factors were antenatal care (ANC) uptake, birth interval, plurality, and place of delivery. Child factors comprised the sex of the child, infant size at birth, birth order, and breastfeeding status. Household factors were wealth status, type of toilet, and source of drinking water [32].

We classified birth interval into two categories: less than 33 months and 33 months or greater based on the World Health Organization (WHO) recommendations on birth spacing [33]. We classified the household’s water source as “improved” if it came from piped water, a tube well, a public tap, a protected spring, a protected dug well, or rainwater, and as “unimproved” if it came from an unprotected spring, an unprotected dug well, a cart with a small tank, vendor-provided water, tanker-truck, or surface water [34]. We categorised the type of toilet facility as “improved” if it was a flush toilet connected to a piped sewer system or septic system, or flush to a pit latrine, a ventilated improved pit latrine, a pit latrine with slab, or composting toilet, and “unimproved” if there is no toilet facility, or if it is a public latrine, a flush toilet discharging elsewhere, a pit latrine without slab, a bucket latrine, or a hanging latrine [34].

### Statistical analysis

We described the background characteristics of the study population using summary tables. A mixed effect robust Poisson regression model was used to identify risk factors of U5M in each time period [35]. First, we performed an unadjusted analysis to identify the association between independent variables and U5M. Variables with a *P*-value of ≤ 0.2 were included in the multivariable-adjusted analysis. We assessed multi-collinearity using the variance inflation factor before the final analysis. To account for random effects, we treated ‘country’ as a random variable and measured it using the intra-class correlation, and the median odds ratio. We calculated the population attributable fraction (PAF), using Levin’s Formula: [36] PAF = P_e_(RR − 1)/[P_e_(RR − 1) + 1], where P_e_ is the prevalence of exposure, and RR is the relative risk associated with the exposure. PAF was computed for each time period. Then, the percentage point change was computed by subtracting the PAF during 1997–2005 from PAF during 2016–2022 to estimate the changes in the contributions of risk/protective factors to U5M over time. The 95% confidence intervals (CIs) for the PAFs were estimated by the Monte Carlo simulation method [37]. We conducted a secondary analysis excluding first births to examine the contribution of the birth interval to U5M. Additionally, sensitivity analyses were conducted based on the 2023/2024 World Bank’s income classification for low-income countries and lower-middle-income countries [27]. Sample weighting was applied to account for survey sample design and non-response.

## Results

### Characteristics of mothers and their births

We included 139,890 live births from 1997 to 2005 and 319,034 from 2016 to 2022. The mean maternal age was 28.4 (± 7.09) years in 1997–2005, and 28.4 (± 6.29) years in 2016–2022. Males comprised 50.8% of under-five children in 1997–2005 and 52.4% in 2016–2022. The proportion of mothers with no education was 51.8% in 1997–2005 but 26.9% in 2016–2020. However, the prevalence of children who never breastfed increased from 3.1% to 6.1% (Table 1).

**Table 1:** Characteristics mothers and births in LLMICs, 1997–2005 and 2016–2022

Variables	1997–2005 (*n* = 139,890) Number (%)	2016–2022 (*n* = 319,034) Number (%)	*P* value (χ^2^ test)
**Maternal socio-demographic characteristics**			
Maternal age (y)			< 0.001
15–19	11,875 (8.49)	15,772 (4.87)	
20–34	98,664 (70.53)	250,738 (77.35)	
35–49	29,351 (20.98)	57,658 (17.79)	
Maternal age at birth (y)			< 0.001
12–19	19,211 (13.73)	29,718 (9.31)	
20–34	97,680 (69.83)	251,392 (78.80)	
35–49	|22,999 (16.44)	37,924 (11.89)	
Age at first birth (y)			< 0.001
< 20	84,977 (60.75)	137,753 (43.18)	
≥ 20	54,913 (39.25)	181,281 (56.82)	
Maternal marital status			< 0.001
Married/in union	128,873 (92.12)	298,436 (93.54)	
Not married/not in union	11,017 (7.88)	20,598 (6.46)	
Maternal educational status			< 0.001
Not educated	72,409 (51.76)	85,834 (26.90)	
Primary	39,950 (28.56)	67,264 (21.09)	
Secondary	21,810 (15.59)	126,936 (39.79)	
Tertiary	5,721 (4.09)	39,000 (12.22)	
Residence			< 0.001
Urban	35,329 (25.25)	102,640 (32.17)	
Rural	104,561 (74.75)	216,394 (67.83)	
**Maternal reproductive and health service-related characteristics**			
Parity			< 0.001
Nulliparous	26,845 (19.19)	88,947 (27.88)	
Two to four	80,705 (57.69)	196,655 (61.64)	
Five or more	32,340 (23.12)	33,432 (10.48)	
Place of delivery			< 0.001
Health facility	51,135 (36.55)	255,078 (79.95)	
Home	88,755 (63.45)	63,956 (20.05)	
Caesarean section delivery	(N = 111,885)		< 0.001
Yes	5,227 (4.67)	52,002 (16.30)	
No	106,658 (95.33)	267,032 (83.70)	
Number of ANC visits			< 0.001
None	39,186 (28.01)	26,994 (8.46)	
1–3 visits	49,456 (35.35)	101,236 (31.73)	
≥ 4 visits	51,248 (36.63)	190,804 (59.81)	
Birth interval	(*N* = 110,020)	(*N* = 228,998)	< 0.001
Less than 33 mon	50,816 (46.19)	98,928 (43.20)	
33 mon and above	59,204 (53.81)	130,070 (56.80)	
Plurality			< 0.001
Yes	2,484 (1.78)	4,453 (1.40)	
No	137,406 (98.22)	314,581 (98.60)	
Child-related characteristics			< 0.001
Sex of child			< 0.001
Male	71,129 (50.85)	167,275 (52.43)	
Female	68,761 (49.15)	151,759 (47.57)	
Size of infant at birth			< 0.001
Very small	16,613 (11.88)	21,762 (6.82)	
Small	19,224 (13.74)	|28,758 (9.01)	
Average	67,148 (48.00)	193,486 (60.65)	
Large	27,718 (19.81)	49,961 (15.66)	
Very large	9,187 (6.57)	25,067 (7.86)	
Birth order			< 0.001
First	29,635 (21.18)	88,947 (27.88)	
Second or higher	110,255 (78.82)	230,086 (72.12)	
Ever breastfed			< 0.001
Yes	135,512 (96.87)	299,472 (93.87)	
No	4,378 (3.13)	19,562 (6.13)	
Household-related characteristics			
Wealth index			< 0.001
Poor	62,291 (44.53)	138,705 (43.48)	
Middle	28,782 (20.58)	62,902 (19.72)	
Rich	48,817 (34.90)	117,427 (36.81)	
Source of drinking water			< 0.001
Improved	76,103 (54.40)	263,026 (82.44)	
Unimproved	63,787 (45.60)	56,008 (17.56)	
Type of toilet			< 0.001
Improved	33,358 (23.85)	205,855 (64.52)	
Unimproved	106,532 (76.15)	113,179 (35.48)	

Wealth index was categorised as poor (merging poorer and poorest), middle, and rich (merging richer and richest)

*ANC* antenatal care

### Changes in under-five mortality rate

In 1997–2005, the U5MR ranged from 40 per 1000 live births in the Philippines to 229 per 1000 live births in Mali. The mean U5MR was 132 (± 48) deaths per 1000 live births. In 2016–2022, the U5MR varied from 16 deaths per 1000 live births in Cambodia to 111 deaths per 1000 live births in Guinea. The mean U5MR was 60 (± 29) deaths per 1000 live births. U5MR showed significant reductions (55.5%) over time, ranging from 20.2% in Madagascar to 87.1% in Cambodia (Supplementary Table S2).

### Contributions of risk/protective factors to U5M in 1997–2005 and 2016–2022

Nine factors had significant contributions to U5M during both time periods. The major contributors to U5M in 1997–2005 were never breastfeeding (PAF = 21.61%, 95% CI 14.95, 27.36), short birth interval (< 33 months) accounted for (PAF 16.24%, 95% CI 12.28, 19.78), and advanced maternal age at birth (PAF = 5.87%, 95% CI 4.16, 7.54). Reductions in U5M were associated with attending at least four ANC visits (PAF = − 11.63%, 95% CI − 14.87%, − 6.98%), one to three ANC visits (PAF = − 8.85%, 95% CI − 12.05%, − 5.85%), and maternal secondary education (PAF = − 5.08%, 95% CI − 6.04%, − 3.73%).

**Fig. 2:**
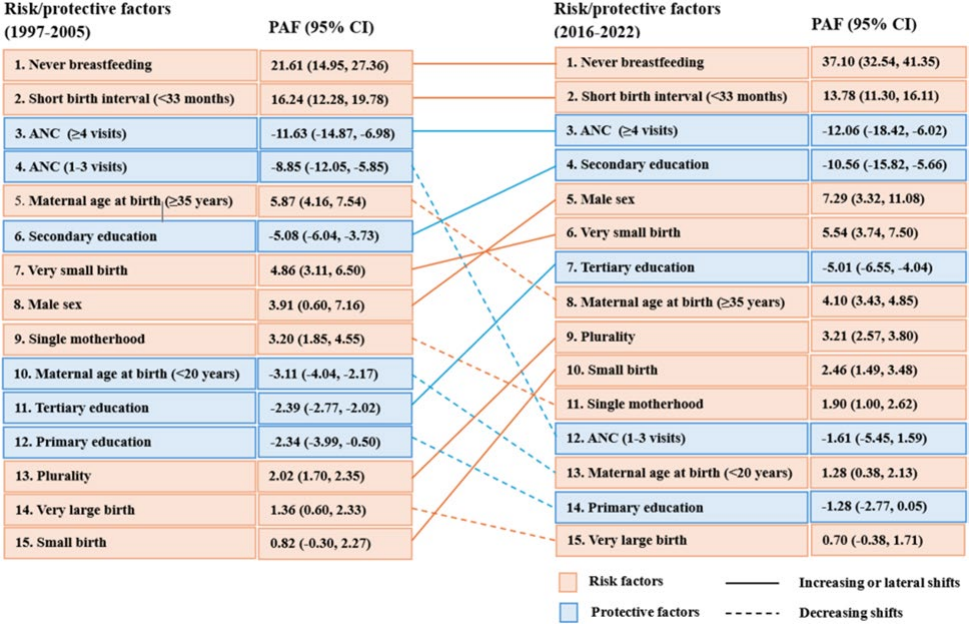
PAFs of risk and protective factors for U5M in LLMICs during 1997–2005 and 2016–2022, and their shifts over time. The risk/protective factors are connected by lines between time periods. *ANC* antenatal care, *CI* confidence interval, *PAF* population attributable fraction

In 2016–2022, never breastfeeding accounted for more than one third of under-five deaths (PAF = 37.10%, 95% CI 32.54%, 41.35%). A short birth interval (< 33 months) accounted for 13.78% of under-five deaths (95% CI 11.30%, 16.11%), while the male sex was associated with 7.29% of under-five deaths (PAF = 7.29%, 95% CI 3.32%, 11.08%). In contrast, attending four or more ANC visits had the greatest protective impact (PAF = − 12.06%, 95% CI − 18.42%, − 6.02%). Similarly, maternal secondary education (PAF = − 10.56%, 95% CI − 15.82%, − 5.66%), and tertiary education (PAF = − 5.01%, 95% CI − 6.55%, − 4.04%) contributed to the second and third highest reduction in U5M (Fig. 2, Supplementary Tables S7–8).

We observed a shift in contributors to U5M over time. In the period 1997–2005, the top five contributors to U5M in descending order were never breastfeeding, ANC (≥ 4 visits), ANC (one to three visits), advanced maternal age at birth (≥ 35 years), and secondary education. In 2016–2022, while the top three contributors to U5M remained the same, the fourth and fifth contributors shifted to secondary education and male sex, respectively (Fig. 2).

### Changes in the contributions of risk factors of U5M from 1997–2005 to 2000–2016

Among the significant contributors to U5M, never breastfeeding, ANC uptake (one to three visits), plurality, maternal age at birth (< 20 years), and maternal education (secondary and tertiary education) showed significant changes in PAF over time. The contribution of never breastfeeding to U5M increased by 15.49 percentage point increase (95% CI 8.31, 23.18). The contribution of secondary education to U5M reduction increased by 5.48 percentage points (95% CI − 11.00, − 0.44). However, the contribution of ANC uptake (one to three visits) to U5M decreased by 7.24 percentage points (95% CI 2.38, 11.69) over the period (Table 2).

**Table 2:** Changes in the contributions of risk factors to U5M in LLMICs, 1997–2005 to 2016–2022

Risk/protective factors	1997–2005	2016–2022	Changes in PAF (percentage points) (95% CI)
	PAF, % (95% CI)	PAF, % (95% CI)	
Maternal age at birth			
12–19	**−** **3.11 (− 4.04, − 2.17)**	**1.28 (0.38, 2.13)**	**5.39 (3.06, 5.65)**
20–34	Ref	Ref	Ref
35–49	**5.87 (4.16, 7.54)**	**4.10 (3.43, 4.85)**	− 1.76 (− 3.72, 0.01)
Marital status			
Married	Ref	Ref	Ref
Not married	**3.20 (1.85, 4.55)**	**1.90 (1.00, 2.62)**	− 1.30 (− 2.91, 0.16)
Maternal educational status			
Not educated	Ref	Ref	Ref
Primary	**− 2.34 (− 3.99, − 0.50)**	− 1.28 (− 2.77, 0.05)	− 1.06 (− 1.36, 3.12)
Secondary	**− 5.08 (− 6.04, − 3.73)**	**− 10.56 (− 15.82, − 5.66)**	**− 5.48 (− 11.00, − 0.44)**
Tertiary	**− 2.39 (− 2.77, − 2.02)**	**− 5.01 (− 6.55, − 4.04)**	**− 2.62 (− 4.17, − 1.65)**
ANC uptake			
None	Ref	Ref	Ref
1–3 visits	**− 8.85 (− 12.05, − 5.85)**	− 1.61 (− 5.45, 1.59)	**7.24 (2.38, 11.69)**
≥ 4 visits	**− 11.63 (− 14.87, − 6.98)**	**− 12.06 (− 18.42, − 6.02)**	− 0.43 (− 9.32, 6.35)
Plurality			
Yes	**2.02 (1.70, 2.35)**	**3.21 (2.57, 3.80)**	**1.19 (0.45, 1.78)**
No	Ref	Ref	Ref
Birth interval			
Less than 33 months	**16.24 (12.28, 19.78)**	**13.78 (11.30, 16.11)**	− 2.46 (− 6.83, 2,13)
33 months and above	Ref	Ref	Ref
Sex of child			
Male	**3.91 (0.60, 7.16)**	**7.29 (3.32, 11.08)**	3.38 (− 2.02, 8.40)
Female	Ref	Ref	Ref
Size of birth at delivery			
Very small	**4.86 (3.11, 6.50)**	**5.54 (3.74, 7.50)**	0.68 (− 1.78, 3.19)
Small	0.82 (− 0.30, 2.27)	**2.46 (1.49, 3.48)**	1.64 (− 0.05, 3.05)
Average	Ref	Ref	Ref
Large	1.56 (− 0.09, 3.03)	− 0.31 (− 1.32, 0.03)	**− **
Very large	**1.36 (0.60, 2.33)**	0.70 (− 0.38, 1.71)	− 0.66 (− 2.22, 0.53)
Ever breastfed			
Yes	Ref	Ref	Ref
No	**21.61 (14.95, 27.36)**	**37.10 (32.54, 41.35)**	**15.49 (8.59, 22.90)**

*ANC* antenatal care, *CI* confidence interval, *PAF* population attributable fraction

Values in bold indicate statistically significant contributions

In our sensitivity analyses based on World Bank income classification, we identified similar contributors to U5M in both LICs and LMICs. The observed changes in the contributions of risk factors to U5M in LMICs were consistent with the main analyses, except for a significant decrease in the contribution of advanced maternal age at birth (≥ 35 years). In LICs, the contribution of ANC uptake (one to three visits) and secondary education did not change significantly over time. In contrast, under-five deaths attributed to small and very small infants at birth increased significantly (Supplementary Tables S9–10).

## Discussion

This study examined how the contributions of risk factors to U5M have changed in LLMICs over the past two decades (1997–2005 to 2016–2022). Significant contributors to U5M included never breastfeeding, short birth intervals (< 33 months), ANC uptake, higher maternal education, male sex, early maternal age at birth (< 20 years), advanced maternal age at birth (≥ 35 years), very small births, plurality, and single motherhood. We observed significant changes in the contributions of risk and protective factors including never breastfeeding, ANC uptake (one to three visits), plurality, maternal age at birth (< 20 years), and maternal education (secondary and tertiary), over the past two decades.

Never having been breastfed was the largest contributor to U5M, accounting for over one-fifth of under-five deaths during 1997–2005 and more than one third of U5M during 2016–2022. This represented a 15.5 percentage-point increase in the contribution to U5M over time. This rise in the contribution may represent the increasing proportion of women who did not breastfeed, which could be attributed to several factors. Notably, the growing marketing and promotion of formula feeding might have influenced women’s breastfeeding behaviour and led to a shift towards formula feeding [38–40]. Working outside the home may be another reason for not having breastfed, which may be due to inadequate workplace support or shorter maternity leave [41–44]. The lack of adequate support and education from health providers may also lead mothers to switch to formula feeding when they face difficulties, such as latching issues and inadequate milk supply. The convenience and flexibility of formula feeding may also lead some mothers to prefer it over breastfeeding [45]. Although further research is needed to fully understand the determinants of never breastfeeding, this finding underscores the urgent need for targeted interventions.

The persistent impact of short birth intervals (< 33 months) on U5M in LLMICs is concerning. Short birth intervals contributed the second highest share of U5M in these countries, accounting for 16.2% of under-five deaths in 1997–2005 and 13.8% in 2016–2022, with no significant decline over 20 years. Despite significant efforts, such as ensuring universal access to sexual and reproductive health services, including family planning, and promoting optimal birth spacing, the impact of short birth intervals on U5M has not significantly decreased over time [4, 33]. While LLMICs made substantial progress in modern contraceptive use over the past two decades [46], nearly half of births (46% in 1997–2005 and 43% in 2016–2022) still had short birth intervals. This persistent trend might be attributed to unequal access to family planning across different populations, method misuse, and early discontinuation [47]. Consistent with this, a systematic review and meta-analysis reported a high prevalence of unintended pregnancies (44.7%) among women using contraceptive methods in low- and middle-income countries [48], highlighting the critical gaps in contraceptive adherence and the quality of family planning service in the region. This finding underscores the need for increased investment in overcoming barriers to optimal birth spacing and for targeted interventions for short-interval pregnancies and births.

Maternal age at birth significantly contributes to U5M. Advanced maternal age at birth (≥ 35 years) contributed to 5.9% of U5M in 1997–2005 and 4.1% of U5M in 2016–2022. U5M associated with advanced maternal age might be due to pregnancy complications, including hypertensive disorders of pregnancy [49, 50], gestational diabetes [49, 50], and placenta previa [49], which can result in preterm births, low birth weight infants, and other adverse birth outcomes, increasing the likelihood of early childhood deaths [49, 51–53]. Advanced maternal age is linked with congenital anomalies and birth defects, which are major causes of child mortality [54]. Advanced age is also associated with chronic diseases including diabetes mellitus, hypertension, and heart diseases, which can complicate pregnancies and childbirths, and increase the risk of U5M. Moreover, older mothers may be less likely to adopt healthy behaviour and utilise maternal and child health services due to sociocultural barriers, including entrenched beliefs and limited access to information and education, which may contribute to poor maternal and child health outcomes. The contribution of advanced maternal age to U5M has not significantly changed over time and requires further targeted interventions.

Although teenage pregnancies are generally associated with increased risks of poor maternal and child health outcomes [55, 56], early maternal age at birth (< 20) was associated with a 3.1% reduction in U5M during the 1997–2005 period. However, this finding requires further investigation. One possible explanation is stronger family and community support for young mothers during childbirth and the postnatal period, which was more common in earlier years. In many settings, young mothers were more likely to give birth in their family home, where extended relatives provide close care, helping mitigate risks associated with early childbearing such as financial instability and limited maternal experience. However, by 2016–2022, despite improvements in maternal and child healthcare and a decline in early childbirths, it still contributed to 1.3% of under-five deaths. These births are often unintended and linked to inadequate healthcare, poor nutrition, and limited social support, all of which negatively impact child health outcomes.

Male sex accounted for 3.9% of under-five deaths in 1997–2005 and 7.3% of deaths in 2016–2022. The increased mortality risk among males may be attributed to their biological susceptibility. While further research is warranted, previous studies have shown an elevated risk of pregnancy complications, including hypertensive disorders of pregnancy, placental abruption, postpartum haemorrhage, and congenital anomalies among male foetuses [57, 58]. Moreover, male neonates are at higher risk of preterm complications and neonatal infections such as neonatal sepsis [59, 60]. Additionally, despite a preference for male children, who are often expected to receive better care and nutrition, a meta-analysis indicated a higher risk of malnutrition among males [61]. Although the contribution of male children to U5M slightly increased due to a higher proportion of male births in the latter period, the change was non-significant, likely due to biological variation.

Plurality was associated with 2% of under-five deaths in 1997–2005 and 3% under-five deaths in 2016–2022. Despite a slight reduction in the prevalence of plurality, its impact on U5M significantly increased over time. Plurality contributes to U5M [24] through several pathways. It is associated with a high rate of preterm births [62] and low birth weight [62, 63], which are the main causes of U5M. Multiple pregnancies are prone to complications such as gestational diabetes [62, 64] and preeclampsia [62], which increase the likelihood of adverse outcomes and child deaths. Additionally, malnutrition [65] and limited access to specialised care [66], such as neonatal intensive care units can lead to poor child health outcomes including mortality. The increase in the contribution of plurality to U5M suggests potential gaps in maternal and child health care services in these regions. Thus, addressing these challenges requires investments in quality antenatal and postnatal care, specialised neonatal services, community-based early detection, and referral for high-risk multiple pregnancies.

Single motherhood was associated with 3.2% of under-five deaths in 1997–2005 and 1.9% in 2016–2022. This might be due to economic disadvantages, limited social support, psychosocial stress, and barriers to accessing maternal and child health services [67–69]. Although, the trend showed a non-significant decrease over time, targeted interventions are required to reduce child deaths associated with single motherhood.

Antenatal care uptake has significantly contributed to the reduction of U5M. In particular, having at least four ANC visits resulted in an 11.6% decrease in U5M in 1997–2005 and a 12.1% decrease in 2016–2022. Although attending one to three visits significantly reduced U5M by 8.8% in the earlier period, it no longer had a significant impact in the more recent period. This aligns with the recent WHO recommendations for at least eight ANC contacts to improve maternal and child health outcomes [70]. However, despite a 20.4% increase in the prevalence of four or more ANC visits over time, the contribution to the reduction in U5M did not show a significant change. This finding suggests the need for renewed efforts to ensure high-quality and comprehensive ANC to further reduce under-five deaths in LLMICs.

Maternal education is also associated with reductions in U5M. The protective effects of higher levels of maternal schooling have significantly increased over time. The impact of secondary education increased from 5.1% in 1997–2005 to 10.6% in 2016–2022 and tertiary education increased from 2.4 to 5.0% in the same period. The greater impact of secondary education was due to its high prevalence among women. The observed increase in U5M reduction associated with higher levels of maternal schooling may have resulted from investments in increasing access to education as part of the MDGs and SDGs [4, 5]. These findings underscore the critical importance of promoting quality secondary and higher education for females as a key strategy to drive reductions in preventable under-five deaths in LLMICs.

This study represents the first comprehensive investigation into the changing contributions and relative importance of a wide range of risk factors for U5M in LLMICs over the past two decades. By examining shifts in PAFs associated with these risk factors from 1997–2005 to 2016–2022, we provided critical insights into how drivers of child mortality have changed over time. We utilised nationally representative DHS data from 24 LLMICs and employed a mixed-effects model to account for the inherent variations across countries. Additionally, we conducted sensitivity analyses based on the 2023/2024 World Bank income classification.

However, the following limitations should be considered. The DHS surveys were conducted at different times across countries, preventing us from capturing a specific point in time to fully track trends over the entire period. To address this, we included only surveys with a minimum 15-year gap between them for each country, enabling us to better assess temporal changes in the contributions of risk factors. Although we incorporated data from the 2016–2022 period to account for the SDG era, the DHS data typically predate by up to five years. As a result, our findings may not have fully captured the impacts of ongoing SDG implementations on U5M.

Moreover, although PAF is a useful metric for estimating the proportion of an outcome that could be prevented by eliminating a given risk factor, it assumes a causal relationship that is difficult to establish in a cross-sectional study design. However, the identified risk factors were consistent with other studies [26, 71–73] in LLMICs. Additionally, we were unable to evaluate the effect of exclusive breastfeeding due to data limitations. While we found a significant contribution of very small births in both time periods, the classification of infant size at birth was subjective, relying on maternal estimation and recall [74]. However, this variable is used as a proxy indicator for birth weight at birth in the context of home births. Furthermore, our findings may be limited by unmeasured factors such as maternal health, nutritional status, pregnancy and childbirth complications, child nutritional status, and vaccinations, emphasising the need for further large-scale prospective studies addressing these gaps. There is also the potential for recall bias in these surveys, particularly for factors such as the size infant at birth, ANC uptake, and birth interval. However, the potential contribution of recall bias to birth intervals is likely minimal, as most mothers remember their children’s birth dates, often associating them with cultural and religious events. Additionally, celebrating birth dates is becoming a common practice in many societies, further reinforcing the recall of birth dates.

As the DHS measures the preceding birth interval by subtracting the date of the previous childbirth from that of the current one, we defined a short birth interval as less than 33 months, accounting for the typical 9-month gestation period and the WHO’s recommendation of at least 24 months spacing between birth and the onset of next pregnancy. However, this categorisation may lead to misclassification, particularly for preterm and post-term births, where the actual gestation duration may vary. Despite these limitations, our study provides robust empirical evidence on the evolving drivers of U5M in LLMICs and offers crucial insights for prioritised and targeted interventions.

In conclusion, the main contributors to U5M were never breastfeeding, short birth intervals (< 33 months), ANC uptake, higher maternal education (secondary and tertiary), advanced maternal age at birth (≥ 35 years), early maternal age at birth (< 20 years), very small infants at birth, male sex, plurality, and single motherhood. The contributions of never breastfeeding, early maternal age at birth (< 20 years), plurality, higher maternal education (secondary and tertiary education), and ANC uptake (one to three visits) significantly changed over time. Therefore, future interventions aimed at reducing U5M need to prioritise the promotion of breastfeeding, maternal education, ANC uptake, and other significant contributors. Prospective studies with comprehensive data on maternal and child health factors would further strengthen the understanding of the complex determinants of U5M in these regions.

## Supplementary Information

Below is the link to the electronic supplementary material.

Supplementary file1 (DOCX 241 KB)

## Data Availability

This study used the Demographic and Health survey datasets of low- and lower-middle-income countries, which are available upon registration at www.measuredhs.com.
