# Supplementary material for: Changes in the contributions of risk factors to under-five mortality in low- and lower-middle-income countries (1997–2022): an analysis of Demographic and Health Survey data
Source: World J Pediatr. 2025 May 10;21(5):502–14. doi: 10.1007/s12519-025-00912-8 (PMC12167716; doi:10.1007/s12519-025-00912-8)

**Changes in the contributions of risk factors to under-five mortality in Low- and Lower-Middle-Income Countries (1997-2022): an analysis of Demographic and Health Survey data**

**Supplementary Tables**

Supplementary Table S1. STROBE Statement checklist of items that should be included in reports of cross-sectional studies

Supplementary Table S2. List of low and lower-middle-income countries’ Demographic and Health Survey data included in the analysis

Supplementary Table S3. Risk factors of under-five mortality in 1997–2005 (n=139,890)

Supplementary Table S4. Risk factors of under-five mortality in 1997–2005 (n=110,120) for a sample excluding first births

Supplementary Table S5. Risk factors of under-five mortality in 2016–2022 (n=319,034)

Supplementary Table S6. Risk factors of under-five mortality in 2016–2022 (n=228,998) for a sample excluding first births

Supplementary Table S7. Population attributable fraction of risk factors for under-five mortality in LLMICs from 1997–2005 (n=139,890) to 2016–2022 (n=319,034)

Supplementary Table S8: Changes in the contributions of risk factors to under-five mortality in LLMICs from 1997–2005 (n=110,120) to 2016–2022 (n=228,998), for a sample excluding first births

Supplementary Table S9. Changes in the contributions of risk factors to under-five mortality in low-income countries from 1997–2005 to 2016–2022

Supplementary Table 10. Changes in the contributions of risk factors to under-five mortality in lower-middle-income countries from 1997–2005 to 2016–2022

Supplementary Table S11. Random effects measures for under-five mortality in 1997–2005 and 2016–2022

Supplementary Text S1. Demographic and Health Survey authorisation letter

**Supplementary Figures**

Supplementary Figure S1: Under-five mortality rate in LLMICs from 1997–2005 to 2016–2022

**Supplementary Table S1.** STROBE Statement—Checklist of items that should be included in reports of cross-sectional studies

|  | **Item No** | **Recommendation** | **Pages** |
| --- | --- | --- | --- |
| **Title and abstract** | 1 | (*a*) Indicate the study’s design with a commonly used term in the title or the abstract | 1 |
|  |  | (*b*) Provide in the abstract an informative and balanced summary of what was done and what was found | 2 |
| **Introduction** | | |  |
| Background/rationale | 2 | Explain the scientific background and rationale for the investigation being reported | 4 |
| Objectives | 3 | State specific objectives, including any prespecified hypotheses | 4 |
| **Methods** | | |  |
| Study design | 4 | Present key elements of study design early in the paper | 5 |
| Setting | 5 | Describe the setting, locations, and relevant dates, including periods of recruitment, exposure, follow-up, and data collection | 5 |
| Participants | 6 | (*a*) Give the eligibility criteria, and the sources and methods of selection of participants | 5 |
| Variables | 7 | Clearly define all outcomes, exposures, predictors, potential confounders, and effect modifiers. Give diagnostic criteria, if applicable | 5 |
| Data sources/ measurement | 8* | For each variable of interest, give sources of data and details of methods of assessment (measurement). Describe comparability of assessment methods if there is more than one group | *6* |
| Bias | 9 | Describe any efforts to address potential sources of bias | 5 |
| Study size | 10 | Explain how the study size was arrived at | 5 |
| Quantitative variables | 11 | Explain how quantitative variables were handled in the analyses. If applicable, describe which groupings were chosen and why | 6 |
| Statistical methods | 12 | (*a*) Describe all statistical methods, including those used to control for confounding | 6 |
|  |  | (*b*) Describe any methods used to examine subgroups and interactions | 6 |
|  |  | (*c*) Explain how missing data were addressed | N/A |
|  |  | (*d*) If applicable, describe analytical methods taking account of sampling strategy | 7 |
|  |  | (*e*) Describe any sensitivity analyses | 7 |
| **Results** | | |  |
| Participants | 13* | (a) Report numbers of individuals at each stage of study—eg numbers potentially eligible, examined for eligibility, confirmed eligible, included in the study, completing follow-up, and analysed | 8 |
|  |  | (b) Give reasons for non-participation at each stage | N/A |
|  |  | (c) Consider use of a flow diagram | N/A |
| Descriptive data | 14* | (a) Give characteristics of study participants (eg demographic, clinical, social) and information on exposures and potential confounders | 7-8 |
|  |  | (b) Indicate number of participants with missing data for each variable of interest | Fig 1. |
| Outcome data | 15* | Report numbers of outcome events or summary measures |  |
| Main results | 16 | (*a*) Give unadjusted estimates and, if applicable, confounder-adjusted estimates and their precision (eg, 95% confidence interval). Make clear which confounders were adjusted for and why they were included | 8 |
|  |  | (*b*) Report category boundaries when continuous variables were categorized | 8 |
|  |  | (*c*) If relevant, consider translating estimates of relative risk into absolute risk for a meaningful time period | 8-9 |
| Other analyses | 17 | Report other analyses done—eg analyses of subgroups and interactions, and sensitivity analyses | 9 |
| **Discussion** | | |  |
| Key results | 18 | Summarise key results with reference to study objectives | 10 |
| Limitations | 19 | Discuss limitations of the study, taking into account sources of potential bias or imprecision. Discuss both direction and magnitude of any potential bias | 10-14 |
| Interpretation | 20 | Give a cautious overall interpretation of results considering objectives, limitations, multiplicity of analyses, results from similar studies, and other relevant evidence | 10-14 |
| Generalisability | 21 | Discuss the generalisability (external validity) of the study results | 10-14 |
| **Other information** | | |  |
| Funding | 22 | Give the source of funding and the role of the funders for the present study and, if applicable, for the original study on which the present article is based | Title page |

**Supplementary Table S2.** List of low-and lower-middle-income countries’ Demographic and Health Survey data included in the analysis

| **List of countries** | **World bank region** | **1997–2005** | **2016–2022** |
| --- | --- | --- | --- |
| Benin | Lower-income countries | Benin, 2001 | Benin, 2017–18 |
| Burkina Faso | Lower-middle-income countries | Burkina Faso, 2003 | Burkina Faso, 2021 |
| Cambodia | Lower-middle income | Cambodia, 2000 | Cambodia, 2021–22 |
| Cameroon | Lower-middle income | Cameroon, 1998 | Cameron, 2018 |
| Cote d'Ivoire | Lower-middle income | Cote d'Ivoire, 1998/99 | Cote d’Ivoire, 2021 |
| Ethiopia | Lower income | Ethiopia, 2000 | Ethiopia, 2016 |
| Ghana | Lower-middle income | Ghana, 2003 | Ghana, 2022 |
| Guinea | Lower income | Guinea, 1999 | Guinea, 2018 |
| Haiti | Lower-middle income | Haiti, 2000 | Haiti, 2016–17 |
| India | Lower-middle income | India, 1998-99 | India, 2019–21 |
| Jordan | Lower-middle income | Jordan, 2002 | Jordan, 2017-18 |
| Kenya | Lower-middle income | Kenya, 2003 | Kenya, 2022 |
| Madagascar | Lower income | Madagascar, 2003-04 | Madagascar, 2021 |
| Mali | Lower income | Mali, 2001 | Mali, 2018 |
| Mauritania | Lower-middle income | Mauritania, 2000-01 | Mauritania, 2019–21 |
| Mozambique | Lower income | Mozambique, 2003 | Mozambique, 2022-23 |
| Nepal | Lower-middle income | Nepal, 2001 | Nepal, 2022 |
| Nigeria | Lower-middle income | Nigeria, 2003 | Nigeria, 2018 |
| Philippines | Lower-middle income | Philippines, 2003 | Philippines, 2022 |
| Rwanda | Lower income | Rwanda, 2000 | Rwanda, 2019–20 |
| Senegal | Lower-middle income | Senegal, 1997 | Senegal, 2019 |
| Tanzania | Lower-middle income | Tanzania, 2003-05 | Tanzania, 2022 |
| Uganda | Lower income | Uganda, 2000-01 | Uganda, 2016 |
| Zambia | Lower-middle income | Zambia, 2001-02 | Zambia, 2018 |

**Supplementary Table S3.** Risk factors of under-five mortality in 1997–2005 (n=139,890)

| **Variables** | **Under-five mortality** | | **Crude RR**  **(95% CI)** | **Adjusted RR**  **(95% CI)** |
| --- | --- | --- | --- | --- |
|  | **Yes (9,712)** | **No (130,178)** |  |  |
| **Maternal age at birth (years)** |  |  |  |  |
| 12–19 | 1,263 (13.00) | 17,947 (13.79) | 0.99 (0.94, 1.04) | **0.78 (0.72, 0.85)** |
| 20–34 | 6,235 (64.20) | 91,446 (70.25) | Ref | Ref |
| 35–49 | 2,214 (22.80) | 20,785 (15.96) | **1.48 (1.34, 1.62)** | **1.38 (1.27, 1. 51)** |
| **Marital status** |  |  |  |  |
| Married/in union | 8,592 (88.47) | 120,282 (92.40) | Ref | Ref |
| Not married/not in union | 1,120 (11.53) | 9,896 (7.60) | **1.50 (1.32, 1.72)** | **1.42 (1.26, 1.61)** |
| **Educational status** |  |  |  |  |
| Not educated | 6,047 (62.26) | 66,362 (50.98) | Ref | Ref |
| Primary | 2,618 (26.96) | 37,331 (28.68) | **0.84 (0.78, 0.90)** | **0.92 (0.86, 0.98)** |
| Secondary | 916 (9.43) | 20,894 (16.05) | **0.60 (0.54, 0.66)** | **0.69 (0.62, 0.76)** |
| Tertiary | 131 (1.35) | 5,590 (4.29) | **0.38 (0.30, 0.47)** | **0.43 (0.35, 0.52)** |
| **Household wealth status** |  |  |  |  |
| Poor | 4,777 (49.19) | 57,513 (44.18) | **1.33 (1.13, 1.57)** | 1.08 (0.97, 1.21) |
| Middle | 2,102 (21.64) | 26,681 (20.50) | **1.26 (1.13, 1.40)** | 1.09 (0.98, 1.21) |
| Rich | 2,833 (29.17) | 45,984 (35.32) | Ref | Ref |
| **Residence** |  |  |  |  |
| Urban | 2,023 (20.83) | 33,306 (25.58) | Ref | Ref |
| Rural | 7,689 (79.17) | 96,872 (74.42) | **1.24 (1.10, 1.39)** | 1.03 (0.91, 1.16) |
| **Place of delivery** |  |  |  |  |
| Health institution | 3,109 (32.01) | 48,026 (36.89) | **0.82 (0.74, 0.90)** | 0.95 (0.88, 1.03) |
| Home | 6,603 (67.99) | 82,152 (63.11) | Ref | Ref |
| **ANC uptake** |  |  |  |  |
| None | 3,641 (37.49) | 35,545 (27.30) | Ref | Ref |
| 1–3 visits | 3,271 (37.68) | 46,185 (35.48) | **0.67 (0.59, 0.76)** | **0.77 (0.70, 0.85)** |
| ≥4 visits | 2,800 (28.83) | 48,448 (37.22) | **0.58 (0.48, 0.70)** | **0.74 (0.66, 0.83)** |
| **Sex of child** |  |  |  |  |
| Male | 5,209 (53.63) | 65,920 (50.64) | **1.12 (1.04, 1.20)** | **1.08 (1.02, 1.15)** |
| Female | 4,503 (46.37) | 64,258 (49.36) | Ref | Ref |
| **Size of infant at birth** |  |  |  |  |
| Very small | 3,834 (39.48) | 63,314 (48.64) | **1.97 (1.62, 2.40)** | **1.43 (1.28, 1.60)** |
| Small | 1,794 (18.47) | 14,819 (11.38) | **1.06 (0.98, 1.35)** | **1.06 (0.98, 1.16)** |
| Average | 1,347 (13.87) | 17,877 (13.73) | Ref | Ref |
| Large | 1,914 (19.71) | 25,804 (19.82) | **1.10 (1.01, 1.20)** | **1.08 (1.01, 1.16)** |
| Very large | 823 (8.47) | 8,364 (6.43) | **1.27 (1.14, 1.43)** | **1.21 (1.08, 1.36)** |
| **Birth order** |  |  |  |  |
| First | 2,296 (23.64) | 27,339 (21.00) | **1.19 (1.08, 1.30)** | **1.40 (1.24, 1.59)** |
| Second or higher | 7,416 (76.36) | 102,839 (79.00) | Ref | Ref |
| **Plurality** |  |  |  |  |
| Yes | 571 (5.88) | 1,913 (1.47) | **3.18 (2.78, 3.64)** | **2.16 (1.98, 2.35)** |
| No | 9,141 (94.12) | 128,265 (98.53) | Ref | Ref |
| **Ever breastfed** |  |  |  |  |
| Yes | 7,351 (75.69) | 128,161 (98.45) | **11.26 (8.32, 15.24)** | **9.81 (7.22, 13.33)** |
| No | 2,361 (24.31) | 2,017 (1.55) | Ref | Ref |
| **Type of toilet** |  |  |  |  |
| Improved | 1,658 (17.07) | 31,700 (24.35) | Ref | Ref |
| Unimproved | 8,054 (82.93) | 98,478 (75.65) | **1.33 (1.12, 1.59)** | 1.06 (0.95, 1.20) |
| **Source of drinking water** |  |  |  |  |
| Improved | 4,758 (48.99) | 71,344 (54.80) | Ref | Ref |
| Unimproved | 4,954 (51.01) | 58,834 (45.20) | **1.09 (1.02, 1.15)** | 1.00 (0.94, 1.05) |
| **World bank region** |  |  |  |  |
| Lower-income countries | 3,793 (39.05) | 43,052 (33.07) | 1.28 (0.94, 1.75) | 1.24 (0.97, 1.59) |
| Lower-middle-income countries | 5,919 (60.95) | 87,126 (66.93) | Ref | Ref |
| **Year of survey** |  |  |  |  |
| 1997-2000 | 5,930 | 72,985 | 1.51 (1.10, 2.27) | 1.19 (0.90, 1.56) |
| 2001-2004 | 3,782 | 57,193 | Ref | Ref |
| *ANC* Antenatal care, *RR* relative risk, *CI* confidence interval, *Ref* Reference | | | | |

**Supplementary Table S4.** Risk factors of under-five mortality in 1997–2005 (n=110,120) for a sample excluding first births

| **Variables** | **Under-five mortality** | | **Crude RR**  **(95% CI)** | **Adjusted RR**  **(95% CI)** |
| --- | --- | --- | --- | --- |
|  | **Yes (7,360)** | **No (102,660)** |  |  |
| **Maternal age at birth (years)** |  |  |  |  |
| 12–19 | 278 (3.78) | 5,175 (5.04) | **0.82 (0.72, 0.93)** | **0.74 (0.66, 0.83)** |
| 20–34 | 4,900 (66.58) | 76,890 (74.90) | Ref | Ref |
| 35-49 | 2,182 (29.64) | 20,595 (20.06) | **1.59 (1.46, 1.73)** | **1.47(1.35, 1.59)** |
| **Marital status** |  |  |  |  |
| Married/in union | 6,693 (90.94) | 96,672 (94.17) | Ref | Ref |
| Not married/not in union | 667 (9.06) | 5,988 (5.83) | **1.56 (1.37, 1.78)** | **1.56 (1.38, 1.76)** |
| **Educational status** |  |  |  |  |
| Not educated | 4,760 (64.67) | 55,888 (54.44) | Ref | Ref |
| Primary | 1,949 (26.48) | 29,332 (28.57) | **0.85 (0.78, 0.93)** | 0.96 (0.88, 1.04) |
| Secondary | 571 (7.76) | 14,066 (13.70) | **0.59 (0.52, 0.69)** | **0.74 (0.64, 0.85)** |
| Tertiary | 80 (1.09) | 3,374 (3.29) | **0.41 (0.33, 0.52)** | **0.50 (0.39, 0.63)** |
| **Wealth status** |  |  |  |  |
| Poor | 3,768 (51.20) | 47,519 (46.29) | **1.32 (1.14, 1.53)** | 1.09 (0.98, 1.22) |
| Middle | 1,574 (21.39) | 21,307 (20.75) | **1.23 (1.13, 1.35)** | 1.06 (0.96, 1.17) |
| Rich | 2,018 (27.42) | 33,834 (32.96) | Ref | Ref |
| **Residence** |  |  |  |  |
| Urban | 1,413 (19.20) | 24,504 (23.87) | Ref | Ref |
| Rural | 5,947 (80.80) | 78,156 (76.13) | **1.43 (1.11, 1.40)** | 1.05 (0.91, 1.21) |
| **Place of delivery** |  |  |  |  |
| Health institution | 2,169 (29.47) | 33,877 (33.00) | **0.86 (0.80, 0.92)** | 1.00 (0.93, 1.08) |
| Home | 5,191 (70.53) | 68,783 (67.00) | Ref | Ref |
| **ANC uptake** |  |  |  |  |
| None | 2,842 (38.61) | 30,177 (29.40) | Ref | Ref |
| 1–3 visits | 2,484 (33.75) | 36,818 (35.86) | **0.68 (0.60, 0.77)** | **0.78 (0.70, 86)** |
| ≥4 visits | 2,033 (27.62) | 35,665 (34.74) | **0.61 (0.52, 0.72)** | **0.78 (0.71, 0.85)** |
| **Birth intervals** |  |  |  |  |
| Less than 33 months | 3,967 (53.90) | 46,849 (45.64) | **1.40 (1.27, 1.53)** | **1.42 (1.31, 1.54)** |
| 33 and more | 3,393 (46.10) | 55,811 (54.36) | Ref | Ref |
| **Sex of child** |  |  |  |  |
| Male | 3,903 (53.03) | 52,018 (50.67) | 1.09 (0.99, 1.19) | 1.08 (0.99, 1.16) |
| Female | 3,457 (46.97) | 50,642 (49.33) | Ref | Ref |
| **Size of infant at birth** |  |  |  |  |
| Very small | 1,344 (18.26) | 11,744 (11.44) | **1.94 (1.55, 2.43)** | **1.46 (1.30, 1.64)** |
| Small | 940 (12.77) | 13,566 (13.21) | **1.15 (0.99, 1.33)** | 1.05 (0.94, 1.16) |
| Average | 2,935 (39.88) | 49,600 (48.31) | Ref | Ref |
| Large | 1,470 (19.97) | 20,792 (20.25) | **1.09 (0.99, 1.19)** | 1.07 (0.99, 1.17) |
| Very large | 671 (9.12) | 6,958 (6.78) | **1.29 (1.13, 1.48)** | 1.24 (1.09, 1.42) |
| **Plurality** |  |  |  |  |
| Yes | 486 (6.60) | 1,672 (1.63) | **3.23 (2.84, 3.68)** | **2.22 (2.00, 2.45)** |
| No | 6,874 (93.40) | 100,988 (98.37) | Ref | Ref |
| **Ever breastfed** |  |  |  |  |
| Yes | 5,739 (77.98) | 101,233 (98.61) | Ref | Ref |
| No | 1,621 (22.02) | 1,427 (1.39) | **11.06 (7.94, 15.40)** | **9.42 (6.76, 13.12)** |
| **Type of toilet** |  |  |  |  |
| Improved | 1,177 (15.99) | 23,097 (22.50) | Ref | Ref |
| Unimproved | 6,183 (84.01) | 79,563 (77.50) | **1.30 (1.10, 1.54)** | 1.04 (0.92, 1.18) |
| **Source of drinking water** |  |  |  |  |
| Improved | 3,467 (47.11) | 54,317 (52.91) | Ref | Ref |
| Unimproved | 3,893 (52.89) | 48,343 (47.09) | **1.10 (1.04, 1.16)** | 1.02 (0.96, 1.08) |
| **World bank region** |  |  |  |  |
| Lower-income countries | 2,948 (40.05) | 35,673 (34.75) | 1.23 (0.89, 1.68) | 1.20 (0.94, 1.53) |
| Lower-middle-income countries | 4,412 (59.95) | 66,987 (65.25) | Ref | Ref |
| **Year of survey** |  |  |  |  |
| 1997-2000 | 4,433 (60.23) | 56,587 (55.12) | 1.49 (1.09, 2.22) | 1.18 (0.90, 1.54) |
| 2001-2004 | 2,927 (39.77) | 46,073 (44.88) | Ref | Ref |
| *ANC* Antenatal care, *RR* relative risk, *CI* confidence interval, *Ref* Reference | | | | |

**Supplementary Table S5.** Risk factors of under-five mortality in 2016–2022 (n=319,034)

| **Variables** | **Under-five mortality** | | **Crude RR**  **(95% CI)** | **Adjusted RR**  **(95% CI)** |
| --- | --- | --- | --- | --- |
|  | **Yes (9,776)** | **No (309,258)** |  |  |
| **Maternal age at birth (years)** |  |  |  |  |
| 12–19 | 1,151 (11.77) | **28,557 (9.24)** | **1.31 (1.18, 1.41)** | **1.14 (1.04, 1.25)** |
| 20–34 | 6,833 (69.90) | 244,559 (79.08) | Ref | Ref |
| 35-49 | 1,792 (18.33) | 36,132 (11.68) | **1.57 (1.49, 1.66)** | **1.36 (1.30, 1.42)** |
| **Marital status** |  |  |  |  |
| Married/in union | 8,839 (90.42) | 289,597 (93.64) | Ref | Ref |
| Not married/not in union | 937 (9.58) | 19,661 (6.36) | **1.41 (1.26, 1.58)** | **1.30 (1.16, 1.43)** |
| **Educational status** |  |  |  |  |
| Not educated | 3,830 (39.18) | 82,005 (26.52) | Ref | Ref |
| Primary | 2,287 (23.39) | 64,977 (21.01) | **0.83 (0.77, 0.90)** | 0.94 (0.88, 1.01) |
| Secondary | 3,047 (31.17) | 123,889 (40.06) | **0.61 (0.53, 0.70)** | **0.76 (0.68, 0.85)** |
| Tertiary | 612 (6.26) | 38,388 (12.41) | **0.41 (0.33, 0.51)** | **0.59 (0.51, 0.69)** |
| **Wealth status** |  |  |  |  |
| Poor | 5,206 (53.25) | 133,499 (43.17) | **1.48 (1.42, 1.54)** | **1.26 (1.09, 1.44)** |
| Middle | 1,863 (19.06) | 61,038 (19.74) | **1.25 (1.20, 1.31)** | **1.11 (1.01, 1.21)** |
| Rich | 2,707 (27.69) | 114,721 (37.10) | Ref | Ref |
| **Residence** |  |  |  |  |
| Urban | 2,578 (26.37) | 100,062 (32.36) | Ref | Ref |
| Rural | 7,198 (73.63) | 209,196 (67.64) | **1.33 (1.24, 1.43)** | 1.06 (0.96, 1.16) |
| **Place of delivery** |  |  |  |  |
| Health institution | 2,169 (22.19) | 33,877 (10.95) | **0. 68 (0.62, 0.75)** | 0.90 (0.85, 0.96) |
| Home | 5,191 (53.10) | 68,783 (22.24) | Ref | Ref |
| **ANC uptake** |  |  |  |  |
| None | 1,581 (16.17) | 25,413 (8.22) | Ref | Ref |
| 1–3 visits | 3,501 (35.81) | 97,735 (31.60) | **0.71 (0.64, 0.79)** | 0.95 (0.85, 1.06) |
| ≥4 visits | 4,694 (48.02) | 186,110 (60.18) | **0.51 (0.44, 0.59)** | **0.82 (0.74, 0.92)** |
| **Sex of child** |  |  |  |  |
| Male | 5,386 (55.09) | 161,888 (52.35) | **1.13 (1.05, 1.20)** | **1.15 (1.07, 1.23)** |
| Female | 4,390 (44.91) | 147,370 (47.65) | Ref | Ref |
| **Size of infant at birth** |  |  |  |  |
| Very large | 844 (8.63) | 24,223 (7.83) | **1.17 (1.08, 1.27)** | 1.09 (0.97, 1.23) |
| Large | 1,394 (14.26) | 48,567 (15.70) | 0.99 (0.93, 1.07) | 0.98 (0.93, 1.03) |
| Average | 4,915 (50.28) | 27,630 (8.93) | Ref | Ref |
| Small | 1,128 (11.54) | 20,267 (6.55) | **1.48 (1.35, 1.58)** | **1.28 (1.17, 1.39)** |
| Very small | 1,495 (15.29) | 188,571 (60.98) | **2.96 (2.16, 4.05)** | **1.86 (1.58, 2.19)** |
| **Birth order** |  |  |  |  |
| First | 2,365 (24.19) | 86,583 (28.00) | **0.89 (0.84, 0.95)** | 1.05 (0.98, 1.12) |
| Second or higher | 7,411 (75.81) | 222,675 (72.00) | Ref | Ref |
| **Plurality** |  |  |  |  |
| Yes | **708 (7.24)** | **3,745 (1.21)** | **5.11 (4.33, 6.04)** | **3.37 (2.97, 3.82)** |
| No | **9,068 (92.76)** | **305,513 (98.79)** | Ref | Ref |
| **Ever breastfed** |  |  |  |  |
| Yes | 5,941 (60.77) | 293,531 (94.91) | Ref | Ref |
| No | 3,835 (39.23) | 15,727 (5.09) | **12.01 (10.09, 14.30)** | **10.62 (8.98, 12.56)** |
| **Type of toilet** |  |  |  |  |
| Improved | 5,305 (54.27) | 200,551 (64.85) | **0.76 (0.67, 0.87)** | 0.99 (0.94, 1.03) |
| Unimproved | 4,471 (45.73) | 108,707 (35.15) | Ref | Ref |
| **Source of drinking water** |  |  |  |  |
| Improved | 7,578 (77.52) | 255,448 (82.60) | **0.90 (0.81, 0.99)** | 1.04 (0.97, 1.11) |
| Unimproved | 2,198 (22.48) | 53,810 (17.40) | Ref | Ref |
| **World bank region** |  |  |  |  |
| Lower-income countries | 2,068 (21.15) | 53,266 (17.22) | **1.33 (0.96, 1.84)** | 1.01 (0.71, 1.45) |
| Lower-middle-income countries | 7,708 (78.85) | 255,992 (82.78) | Ref | Ref |
| **Year of survey** |  |  |  |  |
| 2016-2019 | 8,238 (84.27) | 254,823 (82.40) | **1.42 (1.01, 2.01)** | **1.69 (1.14, 2.50)** |
| 2020-2022 | 1,538 (15.73) | 54,435 (17.60) | Ref | Ref |
| *ANC* Antenatal care, *RR* relative risk, *CI* confidence interval, *Ref* Reference | | | | |

**Supplementary Table S6.** Risk factors of under-five mortality in 2016–2022, (n=228,998) for a sample excluding first births

| **Variables** | **Under-five mortality** | | **Crude RR**  **(95% CI)** | **Adjusted RR**  **(95% CI)** |
| --- | --- | --- | --- | --- |
|  | **Yes (7,277)** | **No (221,721)** |  |  |
| **Maternal age at birth (years)** |  |  |  |  |
| 12–19 | 252 (3.46) | 6,151 (2.78) | 1.23 (0. 98, 1.54) | 0.96 (0.78, 1.20) |
| 20–34 | 5,284 (72.61) | 180,754 (81.52) | Ref | Ref |
| 35-49 | 1,741 (23.93) | 34,816 (15.70) | **1.56 (1.46, 1.66)** | **1.42 (1.35, 1.49)** |
| **Marital status** |  |  |  |  |
| Married/in union | 6,748 (92.73) | 211,403 (95.35) | Ref | Ref |
| Not married/not in union | 529 (7.27) | 10,318 (4.65) | **1.56 (1.37, 1.78)** | **1.37 (1.22, 1.55)** |
| **Educational status** |  |  |  |  |
| Not educated | 3,257 (44.76) | 70,904 (31.98) | Ref | Ref |
| Primary | 1,758 (24.16) | 51,952 (23.43) | **0.82 (0.74, 0.91)** | 0.94 (0.84, 1.02) |
| Secondary | 1,954 (26.85) | 78,885 (35.58) | **0.62 (0.53, 0.73)** | **0.76 (0.66, 0.87)** |
| Tertiary | 308 (4.23) | 19,980 (9.01) | **0.40 (0.30, 0.53)** | **0.54 (0.45, 0.66)** |
| **Wealth status** |  |  |  |  |
| Poor | 3,979 (54.68) | 103,051 (46.48) | **1.52 (1.24, 1.85)** | 1.14 (0.99, 1.31) |
| Middle | 1,405 (19.31) | 43,759 (19.73) | **1.25 (1.14, 1.37)** | 1.06 (0.98, 1.15) |
| Rich | 1,893 (26.01) | 74,911 (33.79) | Ref | Ref |
| **Residence** |  |  |  |  |
| Urban | 1,851 (25.44) | 68,327 (30.82) | Ref | Ref |
| Rural | 5,426 (74.56) | 153,394 (69.18) | **1.31 (1.24, 1.38)** | 1.06 (0.95, 1.18) |
| **Place of delivery** |  |  |  |  |
| Health institution | 4,696 (64.53) | 169,720 (76.55) | **0.72 (0.66, 0.78)** | 0.94 (0.88, 1.01) |
| Home | 2,581 (35.47) | 52,001 (23.45) | Ref | Ref |
| **ANC uptake** |  |  |  |  |
| None | 1,297 (17.82) | 21,123 (9.53) | Ref | Ref |
| 1–3 visits | 2,591 (35.61) | 73,316 (33.07) | **0.72 (0.64, 0.80)** | 0.93 (0.85, 1.03) |
| ≥4 visits | 3,389 (46.57) | 127,282 | **0.54 (0.46, 0.63)** | **0.85 (0.76, 0.94)** |
| **Birth interval** |  |  |  |  |
| Less than 33 months | 3,718 (51.09) | 95,210 | **1.35 (1.28, 1.42)** | **1.36 (1.29, 1.44)** |
| 33 and more | 3,559 (48.91) | 126,511 | Ref | Ref |
| **Sex of child** |  |  |  |  |
| Male | 3,956 (54.36) | 116,285 | **1.09 (0.99, 1.19)** | **1.11 (1.01, 1.22)** |
| Female | 3,321 (45.95) | 105,436 | Ref | Ref |
| **Size of infant at birth** |  |  |  |  |
| Very large | 643 (8.84) | 17,914 (8.08) | **1.15 (1.04, 1.26)** | **1.09 (0.93, 1.29)** |
| Large | 1,053 (14.47) | 36,190 (16.32) | **0.96 (0.88, 1.05)** | 0.94 (0.89, 1.01) |
| Average | 3,645 (50.09) | 133,493 (60.21) | Ref | Ref |
| Small | 1,078 (14.81) | 19,159 (8.64) | **1.52 (1.40, 1.65)** | **1.32 (1.20, 1.45)** |
| Very small | 858 (11.79) | 14,965 (6.75) | **2.79 (2.19, 3.55)** | **1.78 (1.58, 1.99)** |
| **Plurality** |  |  |  |  |
| Yes | 574 (7.89) | 2,791 (1.26) | **5.24 (4.29, 6.41)** | **3.45 (3.00, 3.97)** |
| No | 6,703 (92.11) | 218,930 (98.74) | Ref | Ref |
| **Ever breastfed** |  |  |  |  |
| Yes | 4,568 (62.77) | 210,757 (95.06) | Ref | Ref |
| No | 2,709 (37.23) | 10,964 (4.94) | **11.42 (9.62, 13.56)** | **9.82 (8.22, 11.72)** |
| **Type of toilet** |  |  |  |  |
| Improved | 3,776 (51.89) | 136,792 (61.70) | Ref | Ref |
| Unimproved | 3,501 (48.11) | 84,929 (38.30) | **1.27 (1.13, 1.43)** | 1.01 (0.97, 1.05) |
| **Source of drinking water** |  |  |  |  |
| Improved | 5,574 (76.60) | 179,954 (81.16) | Ref | Ref |
| Unimproved | 1,703 (23.40) | 41,767 (18.84) | 1.08 (0.97, 1.19) | 0.95 (0.88, 1.01) |
| **World bank region** |  |  |  |  |
| Lower-income countries | 1,615 (22.19) | 41,825 (18.86) | 1.28 (0.93, 1.75) | 0.98 (0.69, 1.40) |
| Lower-middle-income countries | 5,662 (77.81) | 179,896 (81.14) | Ref | Ref |
| **Year of survey** |  |  |  |  |
| 2016-2019 | 6,170 (84.79) | 182,071 (82.12) | **1.43 (1.04, 1.96)** | **1.66 (1.15, 2.38)** |
| 2020-2022 | 1,107 (15.21) | 39,650 (17.88) | Ref | Ref |
| *ANC* Antenatal care, *RR* relative risk, *CI* confidence interval,, *Ref* Reference | | | | |

**Supplementary Table S7.** Population attributable fraction of risk factors for under-five mortality in LLMICs from 1997–2005 (n=139,890) to 2016–2022 (n=319,034)

| **Risk/protective factors** | **1997–2005** | | | **2016–2022** | | |
| --- | --- | --- | --- | --- | --- | --- |
|  | **Prevalence of exposure (%) (95% CI)** | **Adjusted RR (95% CI)** | **PAF (%) (95% CI)** | **Prevalence of exposure (%) (95% CI)** | **Adjusted RR (95% CI)** | **PAF (%) (95% CI)** |
| **Maternal age at birth** |  |  |  |  |  |  |
| 12–19 | 13.73 (13.55, 13.91) | **0.78 (0.72, 0.85)** | **-3.11 (-4.04, -2.17)** | 9.31 (9.21, 9.42) | **1.14 (1.04, 1.25)** | **1.28 (0.38, 2.13)** |
| 20–34 | 69.83 (69.58, 70.07) | Ref | Ref | 78.80 (78.66, 78.94) | Ref | Ref |
| 35-49 | 16.44 (16.25, 16.63) | **1.38 (1.27, 1. 51)** | **5.87 (4.16, 7.54)** | 11.89 (11.78, 12.00) | **1.36 (1.30, 1.42)** | **4.10 (3.43, 4.85)** |
| **Marital status** |  |  |  |  |  |  |
| Married | 92.12 (91.98, 92.26) | Ref | Ref | 93.54 (93.46, 93.63) | Ref | Ref |
| Not married | 7.88 (7.73, 8.02) | **1.42 (1.26, 1.61)** | **3.20 (1.85, 4.55)** | 6.46 (6.37, 6.54) | **1.30 (1.16, 1.43)** | **1.90 (1.00, 2.62)** |
| **Maternal educational status** |  |  |  |  |  |  |
| Not educated | 51.76 (51.50, 52.02) | Ref | Ref | 26.91 (26.75, 27.06) | Ref | Ref |
| Primary | 28.56 (28.32, 28.79) | **0.92 (0.86, 0.98)** | **-2.34 (-3.99, -0.50)** | 21.08 (20.94, 21.22) | 0.94 (0.88, 1.01) | -1.28 (-2.77, 0.05) |
| Secondary | 15.59 (15.40, 15.78) | **0.69 (0.62, 0.76)** | **-5.08 (-6.04, -3.73)** | 39.79 (36.62, 39.96) | **0.76 (0.68, 0.85)** | **-10.56 (-15.82, -5.66)** |
| Tertiary | 4.09 (3.99, 4.19) | **0.43 (0.35, 0.52)** | **-2.39 (-2.77, -2.02)** | 12.22 (12.11, 12.34) | **0.59 (0.51, 0.69)** | **-5.01 (-6.55, -4.04)** |
| **ANC uptake** |  |  |  |  |  |  |
| None | 28.02 (27.78, 28.25) | Ref | Ref | 8.46 (8.36, 8.56) | Ref | Ref |
| 1–3 visits | 35.35 (35.38, 35.60) | **0.77 (0.70, 0.85)** | **-8.85 (-12.05, -5.85)** | 31.73 (31.57, 31.89) | 0.95 (0.85, 1.06) | -1.61 (-5.45, 1.59) |
| ≥4 visits | 36.63 (36.38, 36.87) | **0.74 (0.66, 0.83)** | **-11.63 (-14.87, -6.98)** | 59.81 (59.64, 59.98) | **0.82 (0.74, 0.92)** | **-12.06 (-18.42, -6.02)** |
| **Sex of child** |  |  |  |  |  |  |
| Male | 50.85 (50.58, 51.11) | **1.08 (1.02, 1.15)** | **3.91 (0.60, 7.16)** | 52.43 (52.26, 52.60) | **1.15 (1.07, 1.23)** | **7.29 (3.32, 11.08)** |
| Female | 49.15 (48.89, 49.42) | Ref |  | 47.57 (47.39, 47.74) | Ref |  |
| **Size of birth at delivery** |  |  |  |  |  |  |
| Very small | 11.88 (11.71, 12.05) | **1.43 (1.28, 1.60)** | **4.86 (3.11, 6.50)** | 6.82 (6.73, 6.91) | **1.86 (1.58, 2.19)** | **5.54 (3.74, 7.50)** |
| Small | 13.74 (13.56, 13.92) | **1.06 (0.98, 1.16)** | **0.82 (-0.30, 2.27)** | 9.01 (8.91, 9.11) | **1.28 (1.17, 1.39)** | **2.46 (1.49, 3.48)** |
| Average | 48.00 (47.74, 48.26) | Ref | Ref | 60.65 (60.48, 60.82) | Ref | Ref |
| Large | 19.81 (19.60, 20.02) | **1.08 (1.01, 1.16)** | 1.56 (-0.09, 3.03) | 15.66 (15.53, 15.79) | 0.98 (0.93, 1.03) | -0.31 (-1.32, 0.03) |
| Very large | 6.57 (6.44, 6.70) | **1.21 (1.08, 1.36)** | **1.36 (0.60, 2.33)** | 7.86 (7.76, 7.95) | 1.09 (0.97, 1.23) | 0.70 (-0.38, 1.71) |
| **Plurality** |  |  |  |  |  |  |
| Yes | 1.78 (1.71, 1.84) | **2.16 (1.98, 2.35)** | **2.02 (1.70, 2.35)** | 1.40 (1.36, 1.44) | **3.37 (2.97, 3.82)** | **3.21 (2.57, 3.80)** |
| No | 98.22 (98.15, 98.29) | Ref | Ref | 98.60 (98.56, 98.64) | Ref | Ref |
| **Ever breastfed** |  |  |  |  |  |  |
| Yes | 96.87 (96.78, 96.96) | Ref | Ref | 93.87 (93.78, 93.95) | Ref | Ref |
| No | 3.13 (2.66, 2.89) | **9.81 (7.22, 13.33)** | **21.61 (14.95, 27.36)** | 6.13 (6.05, 6.22) | **10.62 (8.98, 12.56)** | **37.10 (32.54, 41.35)** |
| *ANC* Antenatal care, *RR* relative risk, *CI* confidence interval, *PAF* population attributable fraction, *Ref* Reference | | | | | | |

**Supplementary Table S8.** Changes in the contributions of risk factors of under-five mortality in LLMICs from 1997–2005 (n=110,120) to 2016–2022 (n=228,998) for a sample excluding first births

| **Risk/protective factors** | **1997–2005** | | | **2016–2022** | | | **Change in PAF (percentage points) (95% CI)** |
| --- | --- | --- | --- | --- | --- | --- | --- |
|  | **Prevalence of exposure (%) (95% CI)** | **Adjusted RR (95% CI)** | **PAF (%) (95% CI)** | **Prevalence of exposure (%) (95% CI)** | **Adjusted RR (95% CI)** | **PAF (%) (95% CI)** |  |
| **Maternal age at birth** |  |  |  |  |  |  |  |
| 12–19 | 4.96 (4.83, 5.07) | **0.74 (0.66, 0.83)** | **-1.30 (-1.74, -0.87)** | 2.80 (2.73, 2.86) | **0.96 (0.78, 1.20)** | **-0.11 (-0.70, 0.47)** | **1.19 (0.46, 1.92)** |
| 20–34 | 74.34 (74.08, 74.60) | Ref | Ref | 81.24 (81.08, 81.40) | Ref | Ref | Ref |
| 35-49 | 20.70 (20.46, 20.94) | **1.47 (1.35, 1.59)** | **8.86 (6.74, 10.89)** | 15.96 (15.81, 16.11) | **1.42 (1.35, 1.49)** | **6.28 (5.28, 7.25)** | -2.58 (-4.84, 0.26) |
| **Marital status** |  |  |  |  |  |  |  |
| Married | 93.95 (93.77, 94.12) | Ref | Ref | 95.26 (95.15, 95.37) | Ref | Ref | Ref |
| Not married | 6.05 (5.88, 6.22) | **1.56 (1.38, 1.76)** | **3.28 (2.18, 4.34)** | 4.74 (4.63, 4.84) | **1.37 (1.22, 1.55)** | **1.72 (0.97, 2.47)** | **-1.56 (-2.87, -0.23)** |
| **Maternal educational status** |  |  |  |  |  |  |  |
| Not educated | 55.13 (54.77, 55.48) | Ref | Ref | 32.38 (32.14, 32.62) | Ref | Ref | Ref |
| Primary | 28.43 (28.11, 28.76) | 0.96 (0.88, 1.04) | -1.15 (-3.53, 1.11) | 23.45 (23.24, 23.67) | **0.94 (0.84, 1.02)** | -1.44 (-3.67, 0.70) | -0.29 (-3.48, 2.93) |
| Secondary | 13.30 (13.07, 13.54) | **0.74 (0.64, 0.85)** | **-3.58 (-5.10, -2.12)** | 35.31 (35.04, 35.56) | **0.76 (0.66, 0.87)** | **-9.25 (-13.85, -5.02)** | **-5.67 (-10.52, -1.18)** |
| Tertiary | 3.14 (3.02, 3.26) | **0.50 (0.39, 0.63)** | **-1.59 (-2.38, -0.91)** | 8.86 (8.68, 9.04) | **0.54 (0.45, 0.66)** | **-4.25 (-5.27, -3.25)** | **-2.66 (-3.89, -1.39)** |
| **ANC uptake** |  |  |  |  |  |  |  |
| None | 30.01 (29.68, 30.34) | Ref | Ref | 9.79 (9.63, 9.95) | Ref | Ref | Ref |
| 1–3 visits | 35.72 (35.38, 36.06) | **0.78 (0.70, 0.86)** | **-8.53 (-12.54, -5.48)** | 33.15 (32.90, 33.39) | 0.93 (0.85, 1.03) | -2.38 (-5.60, 0.65) | **6.15 (1.82, 11.22)** |
| ≥4 visits | 34.27 (33.93, 34.60) | **0.78 (0.71, 0.85)** | **-8.15 (-11.05, -5.41)** | 57.06 (56.80, 57.32) | **0.85 (0.76, 0.94)** | **-9.36 (-15.84, -3.52)** | -1.21 (-8.32, 5.27) |
| **Plurality** |  |  |  |  |  |  | Ref |
| Yes | 1.96 (1.87, 2.06) | **2.22 (2.00, 2.45)** | **2.33 (1.90, 2.77)** | 1.47 (1.41, 1.53) | **3.45 (3.00, 3.97)** | **3.47 (2.79, 4.15)** | **1.14 (0.33, 1.94)** |
| No | 90.04 (97.94, 98.13) | Ref | Ref | 98.53 (98.47, 98.59) | Ref | Ref | Ref |
| **Birth interval** |  |  |  |  |  |  |  |
| Less than 33 months | 46.18 (45.83, 46.54) | **1.42 (1.31, 1.54)** | **16.24 (12.28, 19.78)** | 43.20 (42.94, 43.46) | **1.36 (1.29, 1.44)** | **13.78 (11.30, 16.11)** | **1.14 (0.33, 1.94)** |
| 33 months and above | 53.81 (53.46, 54.17) | Ref | Ref | 56.80 (56.54, 57.06) | Ref | Ref | Ref |
| **Size of birth at delivery** |  |  |  |  |  |  |  |
| Very small | 11.90 (11.67, 12.12) | **1.46 (1.30, 1.64)** | **5.19 (3.32, 6.98)** | 7.00 (6.87, 7.14) | **1.78 (1.58, 1.99)** | **5.17 (3.87, 6.45)** | 0.02 (-2.73, 0.88) |
| Small | 13.18 (12.94, 13.43) | 1.05 (0.94, 1.16) | **0.65 (-0.79, 2.06)** | 8.74 (8.59, 8.89) | **1.32 (1.20, 1.45)** | **2.72 (1.68, 3.74)** | **2.07 (0.32, 3.84)** |
| Average | 47.75 (47.40, 48.10) | Ref | Ref | 59.89 (59.62, 60.14) | 1 | 1 | Ref |
| Large | 20.24 (19.95, 20.52) | 1.07 (0.99, 1.17) | 1.40 (-0.40, 3.13) | 16.26 (16.07, 16.46) | 0.94 (0.89, 1.01) | -0.98 (-1.99, 0.01) | **-** |
| Very large | 6.93 (6.75, 7.12) | **1.24 (1.09, 1.42)** | **1.63 (0.52, 2.73)** | 8.10 (7.96, 8.25) | **1.09 (0.93, 1.29)** | 0.73 (-0.72, 2.15) | -0.90 (-2.74, 0.87) |
| **Ever breastfed** |  |  |  |  |  |  |  |
| Yes | 97.23 (97.11, 97.34) | Ref | Ref | 94.03 (93.90, 94.16) | Ref | Ref | Ref |
| No | 2.77 (2.66, 2.89) | **9.42 (6.76, 13.12)** | **18.90 (12.64, 24.39)** | 5.97 (5.84, 6.10) | **9.82 (8.22, 11.72)** | **34.50 (29.64, 38.71)** | **15.06 (8.31, 23.18)** |
| *ANC* Antenatal care, *RR* relative risk, *CI* confidence interval, *PAF* population attributable fraction, *Ref* Reference | | | | | | |  |

**Supplementary Table S9.** Changes in the contributions of risk factors of under-five mortality in low-income countries from 1997–2005 to 2016–2022

| **Risk/protective factors** | **1997–2005** | | | **2016–2022** | | | **Change in PAF (percentage points) (95% CI)** |
| --- | --- | --- | --- | --- | --- | --- | --- |
|  | **Prevalence of exposure (%), (95% CI)** | **Adjusted RR (95% CI)** | **PAF (%) (95% CI)** | **Prevalence of exposure (%) (95% CI)** | **Adjusted RR (95% CI)** | **PAF (%) (95% CI)** |  |
| **Maternal age at birth (years)** |  |  |  |  |  |  |  |
| 12–19 | 13.04 (12.74, 13.35) | **0.75 (0.69, 0.92)** | **-3.37 (-4.23, -2.50)** | 13.76 (13.48, 14.05) | **1.31 (1.21, 1.42)** | **4.10 (2.81, 5.30)** | **7.47 (5.94, 8.93)** |
| 20–34 | 66.22 (65.79, 66.64) | Ref | Ref | 67.47 (67.08, 67.86) | Ref | Ref | Ref |
| 35-49 | 20.74 (20.37, 21.10) | **1.35 (1.16, 1.57)** | **6.76 (3.06, 10.22)** | 18.76 (18.44, 19.09) | **1.43 (1.26, 1.61)** | **7.46 (4.61, 10.41)** | 0.70 (-4.35, 5.16) |
| **Maternal marital status** |  |  |  |  |  |  |  |
| Not married | 10.63 (10.36, 10.92) | **1.31 (1.08, 1.58)** | **3.19 (0.56, 5.78)** | 12.76 (12.48, 13.04) | **1.18 (0.93, 1.50)** | **2.24 (0.56, 5.76)** | - 0.95 (-5.33, 2.98) |
| **Maternal educational status** |  |  |  |  |  |  |  |
| Not educated | 64.14 (63.70, 64.57) | Ref | Ref | 40.78 (40.38, 41.19) | Ref | Ref | Ref |
| Primary | 28.55 (28.14, 28.96) | **0.84 (0.79, 0.89)** | **-4.79 (-6.21, -3.17)** | 37.26 (36.86, 37.67) | 1.01 (0.88, 1.14) | 0.37 (-5.02, 4.23) | 5.16 (-0.57, 9.22) |
| Secondary | 6.72 (6.50, 6.95) | 0.63 (0.49, 0.80) | **-2.55 (-3.63, -1.44)** | 18.79 (18.46, 19.11) | 0.93 (0.76, 1.11) | -1.33 (-5.03, 1.84) | 1.22 (-2.65, 4.70) |
| Tertiary | 0.59 (0.52, 0.66) | **0.43 (0.24, 0.76)** | **-0.34 (-0.50, -0.19)** | 3.16 (3.02, 3.31) | **0.60 (0.49, 0.75)** | **-1.28 (-1.68, -0.82)** | **-0.94 (-1.38, -0.48)** |
| **ANC uptake** |  |  |  |  |  |  |  |
| None | 30.99 (30.57, 31.41) | Ref | Ref | 12.39 (12.11, 12.66) | Ref | Ref | Ref |
| 1–3 visits | 38.09 (37.64, 38.52) | **0.83 (0.70, 0.99)** | **-6.92 (-13.58, -0.68)** | 36.26 (35.86, 36.66) | 0.94 (0.73, 1.21) | -2.22 (-12.33, 5.75) | -4.70 (-7.44, 15.04) |
| ≥4 visits | 30.92 (30.51, 31.34) | **0.85 (0.77, 0.94)** | **-4.86 (-8.12, -2.36)** | 51.35 (50.94, 51.77) | 0.80 (0.60, 1.07) | -11.44 (-27.41, 1.64) | -6.58 (-23.48, 7.26) |
| **Sex of the child** |  |  |  |  |  |  |  |
| Male | 50.65 (50.20, 51.10) | **1.09 (1.03, 1.15)** | **4.36 (1.34, 7.36)** | 50.87 (50.45, 51.29) | **1. 26 (1.05, 1.51)** | **11.68 (1.84, 19.71)** | 7.32 (-3.00, 16.46) |
| **Size of infant at birth** |  |  |  |  |  |  |  |
| Very small | 6.79 (6.74, 7.21) | **1.31 (1.07, 1.61)** | **2.06 (0.25, 3.89)** | 9.20 (8.96, 9.44) | **1.84 (1.46, 2.32)** | **7.17 (3.62, 10.20)** | **5.02 (1.10, 8.70)** |
| Small | 15.17 (14.70, 15.34) | 1.01 (0.86, 1.17) | 0.15 (-2.22, 2.61) | 10.59 (10.33, 10.84) | **1.43 (1.29, 1.58)** | **4.36 (2.84, 5.82)** | **4.21 (1.26, 6.93)** |
| Average | 41.02 (40.58, 41.47) | Ref | Ref | 48.38 (47.96, 48.79) | Ref | Ref | Ref |
| Large | 27.13 (26.73, 27.54) | **1.15 (1.03, 1.27)** | **3.91 (0.55, 6.79)** | 20.29 (19.96, 20.63) | 0.98 (0.88, 1.08) | -0.41 (-2.58, 1.62) | **-4.31 (-7.82, -0.60)** |
| Very large | 9.85 (9.58, 10.12) | **1.33 (1.20, 1.47)** | **3.15 (1.88, 4.37)** | 11.55 (11.28, 11.82) | **1.16 (0.98, 1.37)** | 1.81 (-0.47, 3.92) | -1.97 (-3.93, 1.15) |
| **Plurality** |  |  |  |  |  |  |  |
| Yes | 1.73 (1.61, 1.85) | **2.49 (2.29, 2.72)** | **2.51 (2.13, 2.90)** | 1.76 (1.66, 1.88) | **3.68 (2.83, 4.79)** | **4.50 (2.80, 5.98)** | **1.99 (0.23, 3.62)** |
| **Ever breastfed** |  |  |  |  |  |  |  |
| No | 2.39 (2.25, 2.53) | **8.02 (4.55, 14.12)** | **14.35 (5.06, 22.24)** | 8.27 (8.05, 8.50) | **8.98 (5.21, 15.47)** | **39.76 (21.18, 50.88)** | **25.41 (5.58, 39.97)** |
| *ANC* Antenatal care, *RR* relative risk, *CI* confidence interval, *PAF* population attributable fraction, *Ref* Reference | | | | | | | |

**Supplementary Table 10.** Changes in the contributions of risk factors of under-five mortality in lower-middle-income countries from 1997–2005 to 2016–2022

| **Risk/protective factors** | **1997–2005** | | | **2016–2022** | | | **Change in PAF (percentage points) (95% CI)** |
| --- | --- | --- | --- | --- | --- | --- | --- |
|  | **Prevalence of exposure (%), (95% CI)** | **Adjusted RR (95% CI)** | **PAF (%) (95% CI)** | **Prevalence of exposure (%) (95% CI)** | **Adjusted RR (95% CI)** | **PAF (%) (95% CI)** |  |
| **Maternal age at birth** |  |  |  |  |  |  |  |
| 12–19 | 14.08 (13.86, 14.30) | **0.79 (0.70, 0.89)** | **--3.04 (-4.36, -1.74)** | 8.38 (8.28 8.49) | 1.08 (0.99, 1.17) | 0.66 (-0.06, 1.34) | **3.70 (2.18, 5.19)** |
| 20–34 | 71.64 (71.35, 71.93) | Ref | Ref | 81.18 (81.02, 81.32) | Ref | Ref | Ref |
| 35-49 | 14.28 (14.05, 14.50) | **1.42 (1.29, 1.56)** | **5.53 (3.94, 7.31)** | 10.44 (13.95, 14.35) | **1.34 (1.29, 1.40)** | **3.43 (2.89, 4.02)** | **-2.10 (-4.11, -0.46)** |
| **Marital status** |  |  |  |  |  |  |  |
| Not married | 6.49 (6.33, 6.65) | **1.51 (1.29, 1.77)** | **3.20 (1.56, 4.54)** | 5.13 (5.05, 5.22) | **1.35 (1.23, 1.49)** | **1.80 (1.11, 2.37)** | -1.40 (-3.03, 0.23) |
| **Maternal educational status** |  |  |  |  |  |  |  |
| Not educated | 45.53 (45.21, 45.85) | Ref | Ref | 23.99 (23.83, 24.16) | Ref | Ref | Ref |
| Primary | 28.56 (28.27, 28.85) | 0.98 (0.89, 1.07) | -0.57(-2.99, 2.06) | 17.69 (17.54, 17.83) | 0.93 (0.86, 1.00) | -1.25 (-2.51, 0.13) | - |
| Secondary | 20.05 (19.80, 20.31) | **0.75 (0.68, 0.82)** | **-5.28 (-6.81, -3.69)** | 44.19 (44.00, 44.38) | **0.74 (0.68, 0.82)** | **-12.98 (-17.07, -9.09)** | **-7.70 (-12.08, -3.24)** |
| Tertiary | 5.85 (5.70, 6.00) | 0.49 (0.40, 0.60) | **-3.07 (-3.70, -2.49)** | 14.12 (13.99, 14.26) | **0.59 (0.51, 0.69)** | **-6.14 (-7.69, -4.67)** | **-3.07 (-4.70, -1.54)** |
| **ANC uptake** |  |  |  |  |  |  |  |
| None | 2651 (26.23, 26.80) | Ref | Ref | 7.64 (7.54, 7.74) | Ref | Ref | Ref |
| 1–3 visits | 33.98 (33.67, 34.28) | **0.73 (0.68 0.78)** | **-10.10 (-12.18, -7.98 )** | 30.78 (30.61, 30.96) | 0.95 (0.84, 1.07) | -1.56 (-5.40, 1.79) | **8.54 (4.08, 12.55)** |
| ≥4 visits | 39.51 (39.20, 39.82) | **0.68 (0.61, 0.74)** | **-14.47 (-18.21, -11.41)** | 61.58 (61.39, 61.77) | **0.83 (0.73, 0.92)** | **-11.69 (-18.17, -5.55)** | 2.78 (-4.99, 10.48) |
| **Sex of the child** |  |  |  |  |  |  |  |
| Male | 50.94 (50.62, 51.26) | 1.08 (0.98, 1.19) | 3.92 (-1.22, 8.79) | 52.76 (52.56, 52.95) | **1.12 (1.06, 1.18)** | **5.95 (2.96, 8.87)** | 2.03 (-3.78, 8.17) |
| **Size of infant at birth** |  |  |  |  |  |  |  |
| Very small | 14.34 (14.12, 14.57) | **1.50 (1.36, 1.64)** | **6.69 (4.89, 8.39)** | 6.32 (6.23, 6.42) | **1.87 (1.52, 2.30)** | **5.20 (3.08, 7.52)** | 3.70 (-4.21, 1.34) |
| Small | 13.10 (12.88, 13.32) | **1.10 (1.04 1.16)** | **1.29 (0.46, 2.18)** | 8.68 (8.58, 8.79) | **1.24 (1.11, 1.38)** | **2.04 (0.88, 3.23)** | 0.75 (-0.67, 2,19) |
| Average | 51.51 (51.19, 51.83) | Ref | Ref | 63.22 (63.04, 63.41) | Ref | Ref | Ref |
| Large | 16.13 (15.89, 16.37) | 1.01 (0.93, 1.08) | 0.16 (-1.37, 1.22) | 14.69 (14.55, 14.82) | 0.97 (0.92, 1.04) | -0.44 (-1.37, 0.45) | - |
| Very large | 4.91 (4.78, 5.05) | 1.07 (0.91, 1.26) | 0.34 (-0.71, 1.62) | 7.08 (6.98, 7.18) | 1.07 (0.92, 1.23) | 0.49 (-0.07, 1.62) | - |
| **Plurality** |  |  |  |  |  |  |  |
| Yes | 1.80 (1.72, 1.89) | **1.97 (1.81, 2.15)** | **1.72 (1.42, 2.02)** | 1.32 (1.28, 1.36) | **3.27 (2.81, 3.80)** | **2.91 (2.24, 3.50)** | **1.19 (0.46, 1.88)** |
| **Ever breastfed** |  |  |  |  |  |  |  |
| **No** | 3.50 (3.39, 3.62) | **10.87 (8.29, 14.27)** | **25.68 (19.00, 31.42)** | 5.68 (5.59, 5.77) | **11.05 (9.67, 12.62)** | **36.34 (32.85, 39.68)** | **10.66 (4.28, 17.88)** |
| *ANC* Antenatal care, *RR* relative risk, *CI* confidence interval, *PAF* population attributable fraction, *Ref* Reference | | | | | | | |

**Supplementary Table S11.** Random effects measures of under-five mortality in 1997–2005 and 2016–2022

| **Random effects measure** | **1997–2005** | | **2016–2022** | |
| --- | --- | --- | --- | --- |
|  | Null model | Full model | Null model | Full model |
| Variance | 0.20 | 0.12 | 0.20 | 0.18 |
| Intra class correlation Coefficient (ICC) | 5.7% | 3.5% | 5.7% | 5.2% |
| Median odds ratio (MOR) | 1.53 | 1.39 | 1.53 | 1.50 |
| Percent of change in variance (PCV) | - | 40% | - | 10% |

**Supplementary Figure S1.** Under-five mortality rate in LLMICs in 1997–2005 and 2016–2022

Supplementary Text S1: Demographic and Health Survey authorisation letter


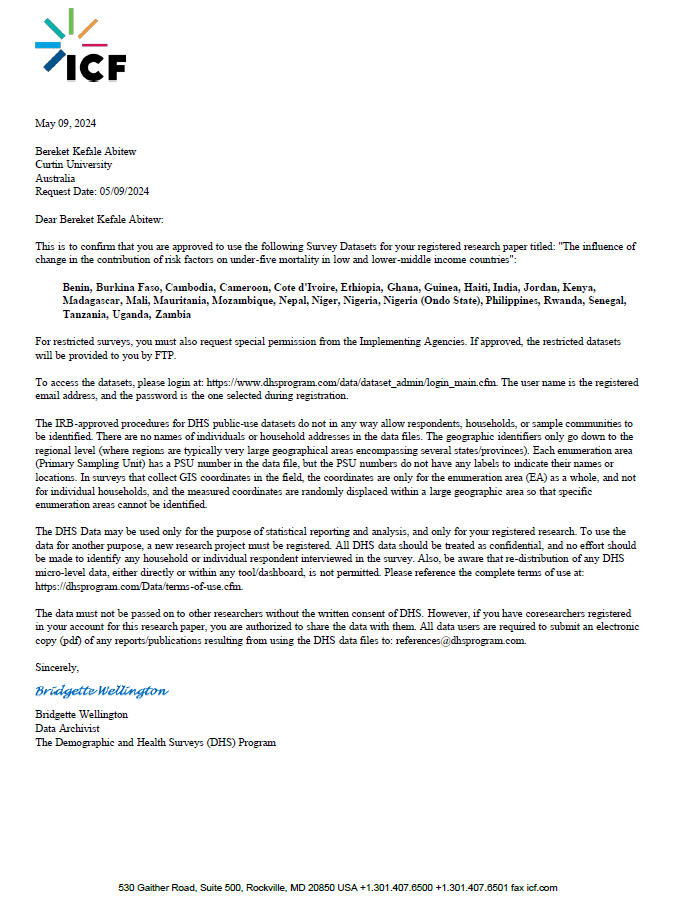

Supplement: Supplementary file 1 — Supplementary file1 (DOCX 241 KB) [file 12519_2025_912_MOESM1_ESM.docx]
